# Blinatumomab in Children with MRD-Positive B-Cell Precursor Acute Lymphoblastic Leukemia: A Report of 11 Cases

**DOI:** 10.3390/hematolrep16020035

**Published:** 2024-06-03

**Authors:** Yi-Lun Wang, Tsung-Yen Chang, Yu-Chuan Wen, Shu-Ho Yang, Yi-Wen Hsiao, Chia-Chi Chiu, Yu-Chieh Chen, Ruei-Shan Hu, Shih-Hsiang Chen, Tang-Her Jaing, Chih-Cheng Hsiao

**Affiliations:** 1 Department of Pediatrics, Division of Hematology/Oncology, Chang Gung Memorial Hospital, Taoyuan 33315, Taiwan; mp2601@cgmh.org.tw (Y.-L.W.); gisborne@cgmh.org.tw (T.-Y.C.); samechen@cgmh.org.tw (S.-H.C.); 2 Department of Nursing, Chang Gung Memorial Hospital, Taoyuan 33315, Taiwan; k22108@cgmh.org.tw (Y.-C.W.); sugar1461@cgmh.org.tw (S.-H.Y.); apple80168@cgmh.org.tw (Y.-W.H.); chi0105@cgmh.org.tw (C.-C.C.); 3 Department of Pediatrics, Kaohsiung Chang Gung Memorial Hospital, Kaohsiung 83301, Taiwan; gesicht27@cgmh.org.tw (Y.-C.C.); hsiaojc@cgmh.org.tw (C.-C.H.); 4 College of Medicine, Chang Gung University, Taoyuan 33302, Taiwan; b070217@cgu.edu.tw

**Keywords:** blinatumomab, measurable residual disease, acute lymphoblastic leukemia, hematopoietic cell transplantation, donor lymphocyte infusion

## Abstract

**Background/Objectives:** Relapsed B-cell acute lymphoblastic leukemia (B-ALL) remains an unresolved matter of concern regarding adverse outcomes. This case study aimed to evaluate the effectiveness of blinatumomab, with or without door lymphocyte infusion (DLI), in treating measurable residual disease (MRD)-positive B-ALL. **Methods:** All patients who received blinatumomab salvage therapy were included in this study. Eleven patients were included in the study. All patients were evaluated for MRD-negativity. **Results:** Before starting blinatumomab therapy, seven patients tested positive for MRD, three tested negative, and one had refractory disease. Hematopoietic cell transplantation (HCT) was reserved for five patients with persistent MRD. Six patients became MRD-negative and subsequent HCT was not performed. Only two patients relapsed; one patient died of relapse, and the other one received carfilzomib-based therapy and was MRD-negative thereafter. Nine patients were MRD-negative at a median follow-up of 28 months (15–52 months). Two of three MRD-positive post-transplant patients remained in complete molecular remission after preemptive DLI at the last follow-up date. In the first salvage, blinatumomab may achieve complete remission and bridging to HCT in pediatric patients with end-of-induction MRD-positive B-cell precursor ALL. **Conclusions:** The decision on how to treat post-transplant relapse continues to affect survival outcomes. Blinatumomab combined with DLI may extend the armamentarium of release options for high-risk pediatric patients. This approach is encouraging for high-risk ALL patients who are MRD-positive post-transplantation.

## 1. Introduction

Despite significant progress in detecting and treating childhood acute lymphoblastic leukemia (ALL), outcomes after relapse remain poor [1]. Immune escape has emerged as an insuperable obstacle to long-term survival [2]. The presence of measurable residual disease (MRD) in B-cell precursor ALL prognosticates the survival of relapsed patients, with a subsequent relapse rate of 90% [3]. On March 29, 2018, the Food and Drug Administration (FDA) granted accelerated approval for blinatumomab for the treatment of adults and children with BCP-ALL in first or second CR with MRD > 0.1% [4,5,6,7]. In December 2020, National Health Insurance (NHI) in Taiwan issued the extension of indication for blinatumomab to include treating children with MRD-positive B-cell precursor ALL as a bridging tool to hematopoietic cell transplantation (HCT). 

Children with refractory or relapsed ALL can benefit from novel immune-targeted approaches. Blinatumomab was manufactured to recognize CD19 expression in B-ALL. Engagement of CD19 expressed by B cells and CD3 on the patient’s cytotoxic T cells leads to the T cell-dependent lysis of CD19-positive leukemia cells. This study aims to explore whether blinatumomab immunotherapy can eliminate the need for HCT in pediatric patients. In addition, patients who tested positive for MRD after HCT may benefit from DLI.

## 2. Materials and Methods

This retrospective observational study included patients with MRD-positive B-cell precursor (BCP)-ALL aged 3–19 years treated at the Chang Gung Memorial Hospital. Patients with MRD ≥ 0.01% at the end of induction (EOI) chemotherapy were classified as MRD high-risk and eligible for treatment with blinatumomab (Amgen, Thousand Oaks, CA, USA). MRD detection was identified using multiparameter flow cytometry and real-time quantitative PCR (RQ-PCR) to predict relapse and leukemia-free survival in ALL. The use of blinatumomab in MRD-positive B-ALL patients needs to be approved by the NHI Supervisory Committee. Each treatment cycle consisted of 4 weeks of continuous intravenous infusion of blinatumomab, followed by a 2-week treatment-free interval. All patients underwent two cycles of blinatumomab to induce durable molecular remission. Written informed consent for data collection and analysis was obtained from the patient for cancer treatment. 

Diagnostic samples were collected from all 11 patients at various stages of their treatment, including the end of induction, consolidation therapy, continuation therapy, before transplant, and post-transplant. Additionally, samples were obtained following treatment after the first bone marrow relapse. BM mononucleated cells were acquired through Ficoll–Hypaque density gradient centrifugation (General Electric, Wauwatosa, WI, USA). A portion of cells were promptly prepared for flow cytometric analysis, while another portion was preserved in Roswell Park Memorial Institute medium with 10% dimethylsulfoxide and 10% fetal bovine serum in liquid nitrogen until the testing time using RQ-PCR.

### 2.1. MRD Detection by RQ-PCR

The RQ-PCR experiment was conducted following the methods described in previous studies [8,9]. RNA was extracted from mononucleated cells using Trizol reagent (Invitrogen Corporation, Carlsbad, CA, USA), followed by synthesis of complementary DNA using the Superscript II RNase H2 reverse transcriptase kit (Invitrogen Corporation). The primers and probes for the three major fusion transcripts were designed based on the specifications provided by the Europe Against Cancer Program. RQ-PCR was conducted using the TaqMan assay to measure the three primary fusion transcripts. The ABL1 gene served as the internal control, and the ABI 7900 system (Applied Biosystems, Foster City, CA, USA) was utilized for this purpose. The transcript amount was quantified as normalized copy number (NCN), which represents the ratio of fusion transcript copy number to that of the ABL1 control gene. Excluded from this study were samples with less than 2 × 10^4^ copies of the ABL1 control gene. The MRD value was calculated as a percentage by dividing the follow-up NCN by the corresponding diagnosis NCN. In our laboratory, the median (ranges) of NCN at diagnosis for the ETV6-RUNX1, TCF3-PBX1, P190 BCR-ABL1, and P210 BCR-ABL1 were 1.59 (0.02–8.16), 5.95 (0.54–25.95), 0.93 (0.10–3.20), and 0.90 (0.67–1.68), respectively. For each run, a sensitivity analysis of the RQ-PCR assay was conducted using serial dilutions of RNAs from K562 cells (specifically for P210 BCR-ABL1) and a P190 BCR-ABL1 cell line. These dilutions were mixed with RNA from HL-60 cells (ranging from 10^−1^ to 10^−6^) or ETV6-RUNX1 and TCF3-PBX1 plasmids (ranging from 5 to 10^6^ copies). The purpose of this analysis was to construct a standard curve and determine the assay’s sensitivity. The sensitivity of detecting P210 BCR-ABL1 and P190 BCR-ABL1 was 10^−5^ for RNAs obtained from cell lines, while for ETV6-RUNX1 or TCF3-PBX1 plasmids, the sensitivity was five copies.

### 2.2. MRD Detection by Flow Cytometry

The mononucleated cells were diluted to a final concentration of 2 × 10^7^/mL. The markers specific to leukemia were identified during diagnosis using a set of six antibodies that were adapted from a previous study [10]. The antibodies were acquired from Becton Dickinson (San Jose, CA, USA) or Beckman Coulter (Brea, CA, USA). The visualization of antibody binding was conducted using a BD FACS Aria III flow cytometer, and subsequent data analysis was performed using FACSDiva Software (Becton Dickinson). The experimental procedures, including cell staining, flow cytometric analysis, and MRD estimates, were conducted following established protocols [10]. Markers specific to leukemia were identified from diagnostic samples. The majority of the blasts in the diagnostic samples analyzed exhibited leukemia-specific markers, with a median of 97.7% and a range of 75–100%. The chosen markers specific to leukemia were subsequently utilized to determine the proportion of remaining leukemia cells in the subsequent samples. The exclusion criteria for this study involved the removal of follow-up samples with <10^5^ mononucleated cells. The MRD value was calculated by dividing the percentage of cells carrying leukemia-specific markers by the total number of mononucleated cells obtained in the follow-up samples. The sensitivity of the detection using six-color flow cytometry was 10^−4^. MRD positivity was determined by the presence of at least 10 cells expressing a leukemia-specific immunophenotype out of 100,000 bone marrow mononucleated cells (equivalent to 0.01% or more).

The detailed strategy for blinatumomab is shown in Supplementary File S1. Therefore, blinatumomab treatment in MRD+ disease followed the Taiwan Pediatric Oncology Group protocol definitions. The 0.01% threshold is utilized to establish MRD-positivity since it corresponds to the standard limit of detection for regular flow cytometric and molecular tests. Complete response (CR) was defined as less than 5% blasts in the bone marrow with no extramedullary disease associated with peripheral hematologic recovery. MRD response was defined as CR without detectable disease, irrespective of the marker or technique employed. 

Adverse events (AE) were recorded and subsequently classified according to the Common Terminology Criteria for AE. Graft-versus-host disease (GVHD) was recorded using the modified Glucksberg criteria for acute GVHD and the 2014 revised National Institutes of Health Consensus Conference Criteria for Chronic GVHD. 

Using the Mann–Whitney U-test, we compared patient age, biological data (weight, height, and body surface area), and clinical laboratory data. Other controllable risk factors in the clinical data were analyzed using Fisher’s exact test and logistic regression analysis. 

## 3. Case Presentation

**Figure 1:**
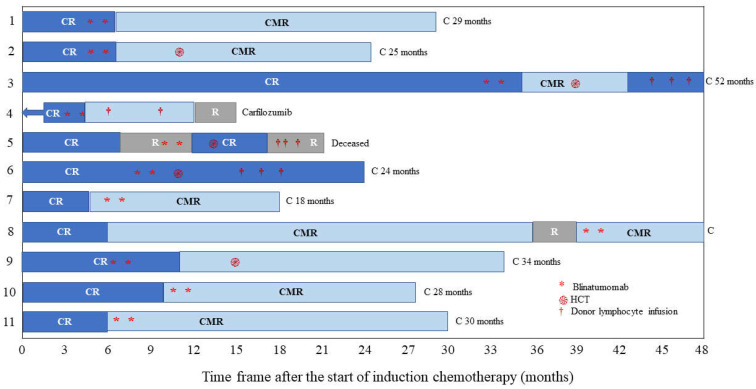
Swimmer’s plot depicts individual patient responses, subsequent therapy, and outcomes for the 11 patients. Abbreviations: C, censored; CMR, complete molecular remission; CR, complete response with or without count recovery; R, relapse; HCT, hematopoietic cell transplantation.

Eleven patients received blinatumomab between July 2021 and December 2022, intending to achieve sustained MRD negativity. Patients received blinatumomab via continuous IV infusions at a flat dose of 15 µg/m^2^/day. At the time of blinatumomab treatment, the study population had a median age of 11.5 years (range 3–19 years), and a wide range of cytogenetic abnormalities were observed in the cohort (Table 1). Figure 1 displays the therapy responses and outcomes for each patient.

Four of the eleven patients were MRD-negative following blinatumomab therapy and subsequent HCT. One patient was MRD-positive on the HCT. One patient experienced a frank relapse before receiving blinatumomab as salvage therapy. She developed grade 2 cytokine release syndrome (CRS) during blinatumomab infusion. Her symptoms resolved with dexamethasone and low-flow oxygen supplementation for one week, but she ultimately succumbed to leukemia relapse 11 months later.

Nine patients achieved CR, with mild toxicity and no grade 3–4 CRS or neurotoxicity. At a median follow-up of 28 months from diagnosis (range, 15–52 months), all patients were evaluable for MRD-negativity; nine patients (82%) were MRD-negative at the first time point.

Therapy-induced depletion of CD19-positive B cells and plasma cells may lead to prolonged hypogammaglobulinemia. In this study, five patients presented with hypogammaglobulinemia secondary to blinatumomab immunotherapy. Four patients received donor lymphocyte infusions (DLI) as a treatment or preventive measure following HCT if MRD-positive status persisted. The median number of DLI cycles and infused CD3+ cell dose was three and 2.2 × 10^7^/kg, respectively. Two of these patients subsequently experienced subsequent relapse and became refractory to multiple therapies.

## 4. Discussion

HCT is currently FDA-approved for the treatment of adult and pediatric BCP-ALL in first or second complete remission with MRD ≥ 0.1% [11,12]. However, HCT continues offering an indisputable therapeutic option for R/R BCP-ALL in children and adolescents. The reported relapse-free survival (RFS) rate after HCT tends to be higher when performed in the first CR [13]. 

Considering that children with ALL Ph+ were an independent predictor for worse prognosis, all children were classified into the high-risk group. According to the EsPhALL2010 protocol, the 5-year overall and event-free survivals were 71.8% and 57.0%, respectively [14]. As an MRD level > 0.01% is associated with a significantly higher risk of relapse after HCT, pre-transplant reduction of disease burden to the lowest possible MRD is pivotal [15]. It is important to highlight that for the proposed indication, neither a complete MRD response nor hematologic RFS alone in a single-arm trial would be sufficient to support approval [7]. 

Blinatumomab is a landmark treatment for B-cell ALL with approval in R/R- and MRD-positive settings [7,16,17]. Patients with MRD > 0.01% at the end of consolidation (EOC) should receive blinatumomab or re-intensification chemotherapy to attain MRD negativity and should be considered for HCT as consolidation therapy. Moreover, it is not advisable to administer intrathecal chemotherapy concurrently with blinatumomab treatment due to an increased risk of neurotoxicity [18]. In a phase III trial, secondary hypogammaglobulinemia was recently reported in 16% of blinatumomab-treated patients with R/R ALL compared to 1% in patients who received chemotherapy [11]. A reduction in IgG levels post-blinatumomab was also observed in patients with ALL who achieved treatment response but not in patients who did not respond to blinatumomab, suggesting an important correlation between the development of secondary hypogammaglobulinemia and blinatumomab treatment response [19]. 

However, we did not determine the relationship between hypogammaglobulinemia and the treatment response in our small series. In this retrospective observational study, the hypothesis that blinatumomab combined with DLI is an alternative strategy should be empirically testable. Including DLI between one month before and 100 days after the initiation of blinatumomab appears to be a safe approach, but it does not appear to enhance outcomes in patients with B-ALL who have experienced relapse following HCT [20]. A careful hypothesis evaluation may be carried out with more comprehensive data. It could be beneficial to expand on the selection criteria for patients and address any potential biases that may have been introduced by the retrospective nature of the study. This combination may increase the anti-leukemic activity of both therapeutic measures and overcome the limitations related to partial T-cell reconstitution after HCT [21,22]. Treatments other than blinatumomab administered post-salvage were heterogeneous [23]. Patients experienced GVHD following DLI but it was manageable. 

## 5. Conclusions

Children who had MRD-positive ALL before HCT might be treated effectively with blinatumomab to delay or avoid HCT. Moreover, post-transplant therapy was not uniform for persistent MRD-positive patients, and even minor improvements could be encouraged. We hypothesize that the combination of blinatumomab and DLI could potentially eliminate the leukemic cells. Due to the restricted sample size, the statistical power of the analysis could be constrained, consequently restricting generalizability. We plan to broaden this study by incorporating a larger number of patients and presenting the statistical significance of the described findings. 

## Figures and Tables

**Table 1:** Patients, disease, and HCT characteristics.

Patient and Disease Characteristics			Blinatumomab Treatment		HCT Characteristics		Outcomes
Age, y/Sex	Cytogenetics	Disease Status before HCT	CRS Grading	Days to HCT	Preparative Regimen	Graft Source	Status
3/F	NA	MRD+	Gr 1	NA	NA	NA	CR1 MRD−
14/F	DUX4r	MRD+	No	165	CyBu	MUD	CR1 MRD−
19/F	Complex karyotype	MRD+	Gr 1	157	CyBu	MUD	CR1 MRD−
18/F	TCF3-ZNF384	R/R	Gr 1	NA	NA	NA	CR3 MRD−
12/F	E2A-PBX1	MRD+	Gr 2	97	CyBu	MSD	DOD
7/F	Ph’(+)	MRD+	Gr 1	125	CyBu	MUD	MRD+
8/M	Ph’(+)	MRD−	No	NA	NA	NA	CR1 MRD−
11.5/M	E2A-PBX1	CR2 MRD−	Gr 1	NA	NA	NA	CR2 MRD−
12/F	JAK2	MRD+	Gr 1	270	CyBu	MUD	CR1 MRD−
4/M	Ph’(+)	MRD−	Gr 2	NA	NA	NA	CR1 MRD−
11/M	DUX4r	MRD+	Gr 1	NA	NA	NA	CR1 MRD−

Abbreviations: CR, complete remission; CRS, cytokine release syndrome; DOD, died of disease; F, female; HCT, hematopoietic cell transplantation; M, male; MRD+, measurable residual disease positive; MRD−, measurable residual disease negative; MUD, matched unrelated donor; NA, not applicable; Ph’, Philadelphia chromosome; R/R, relapsed/refractory.

## Data Availability

The data used in this study are available from the corresponding author upon request.

## Supplementary Materials

The following supporting information can be downloaded at: https://www.mdpi.com/article/10.3390/hematolrep16020035/s1, Scheme S1: Protocols for childhood acute lymphoblastic leukemia.
