# Supplementary material for: Blinatumomab in Children with MRD-Positive B-Cell Precursor Acute Lymphoblastic Leukemia: A Report of 11 Cases
Source: Hematol Rep. 2024 Jun 3;16(2):347–53. doi: 10.3390/hematolrep16020035 (PMC11204057; doi:10.3390/hematolrep16020035)
Supplement: Supplementary file 1 [file hematolrep-16-00035-s001.zip › hematolrep-2576474-supplementary.pdf]

TPOG ALL-2013

PROTOCOLS FOR CHILDHOOD ACUTE LYMPHOBLASTIC LEUKEMIA

FOR

CHILDHOOD CANCER FOUNDATION

OF TAIWAN

BY

TAIWAN PEDIATRIC ONCOLOGY GROUP

Jan. 2013

Contents

1	SYNOPSIS	P.3
2	RISK CLASSIFICATION	P.4
3	CRITERIA FOR STANDARD RISK, HIGH RISK, AND VERY HIGH RISK ALL	P.4
4	TREATMENT PLANS	P.5
4.1	INDUCTION	P.5
4.2	IT CHEMOTHERAPY DURING INDUCTION TREATMENT	P.8
4.3	CONSOLIDATION TREATMENT	P.10
4.4	REINTENSIFICATION TREATMENT	P.12
4.5	CONTINUATION AND REINDUCTION TREATMENT	P.14
4.6	IT CHEMOTHERAPY DURING CONTINUATION TREATMENT	P.17
5	DRUG INFORMATION	P.19
6	TREATMENT MODIFICATIONS	P.28
7	DOSE MODIFICATIONS DURING CONTINUATION THERAPY	P.32
8	PATIENT EVALUATION	P.34
9	CONTINGENCY PLANS FOR REFRACTORY DISEASE OR RELAPSE	P.37
10	SUPPORTIVE CARE	P.39
11	TOXICITY AND COMPLICATIONS CRITERIA	P.42
12	DROP OFF CRITERIA	P.43
13	REFERENCES	P.44-57

1. SYNOPSIS

TPOG has improved the treatment results on childhood acute lymphoblastic leukemia (ALL). St. Jude Total 15 protocol has achieved the most successful results in the world (Pui et al, N Engl J Med 2009). Prof. Ching-Hon Pui, our advisor on ALL protocols, has kindly guided us to adopt Total 15 and 16 for the use of new TPOG ALL protocol. In this protocol, risk classicification will only the determined by the level of minimal residual disease (MRD) at the end of induction therapy. MRD level will be assayed for follow-up. We then have to rely on accurate MRD assay. Leunase 5000 U/m^2 will be increased to 6000 U/m^2 according to the Japanese experience.

We are grateful to Prof.Ching-Hon Pui for his continuous support and guidance for the improvement on treatment results for the children with ALL in Taiwan.

2. RISK CLASSIFICATION

Patients are classified into one of three categories (standard-, high-, or very high-risk) based on the presenting age, leukocyte count, presence or absence of CNS-3 status or testicular leukemia, immunophenotype, cytogenetics and molecular genetics, DNA index, and early response to therapy. Hence, definitive risk assignment will be made after completion of remission induction therapy. The criteria and the estimated proportion of patients in each category are provided below.

3. CRITERIA FOR STANDARD RISK ALL

1.B-lymphoblastic ALL with DNA index ≥ 1.16, *TEL-AML*1 fusion, or age 1 to 9.9 years and presenting WBC < 50,000/mm^3.

2. Must not have:

-CNS 3 status (≥ 5 WBC/μL of cerebrospinal fluid with morphologically identifiable blasts or cranial nerve palsy).

-Overt testicular leukemia (evidenced by ultrasonogram).

-Adverse genetic features: t(9;22) or *BCR-ABL1* fusion; t(1;19) with *E2A-PBX1* fusion; rearranged *MLL* (as measured by FISH and/or PCR); or hypodiploidy (< 44 chromosomes).

-Poor early response (≥ 1% lymphoblasts on day 15 of remission induction, ≥ 0.01% lymphoblasts by immunologic or molecular methods on remission date).

CRITERIA FOR HIGH-RISK ALL

1. All cases of T-cell ALL and those of B- lymphoblastic ALL that do not meet the criteria for standard-risk or very high-risk ALL.

CRITERIA FOR VERY HIGH-RISK ALL

1. t(9;22) or *BCR-ABL1* fusion (with MRD ≥0.01% after remission induction including dasatinib (60 mg/m^2 per day).
2. Infants with t(4;11) or *MLL* fusion.
3. Induction failure or > 1% leukemic lymphoblasts in the bone marrow on remission date (with the exception of hyperdiploid >50 and *TEL-AML1* cases who should have positive MRD after consolidation therapy).
4. > 0.1% leukemic lymphoblasts in the bone marrow in week 7 of continuation treatment (i.e. before reinduction I, ~14 weeks post remission induction).
5. Re-emergence of leukemic lymphoblasts by MRD (at any level) in patients previously MRD negative.
6. Persistently detectable MRD at lower levels.
7. Early T-cell precursor (ETP) ALL, defined by lack of expression of CD1a and CD8 and low or absent expression of CD5 together with aberrant expression of myeloid and

hematopoietic stem cell markers (such as CD13, CD33, CD34 and CD117).

4.TREATMENT PLANS

4.1 Induction (6-7 weeks)

Induction treatment will begin with prednisone, vincristine, epirubicin, L-asparaginase and triple intrathecal treatment, followed by cyclophosphamide plus cytarabine plus mercaptopurine. Epirubicin may be delayed in patients with febrile neutropenia, evidence of mucositis or increased hyperbilirubinemia (i.e., total bilirubin ≥ 2.0 mg/dl and direct bilirubin > 1.4 mg/dl). Patients with mucositis should be evaluated for herpes simplex infection and treated with acyclovir if work-up is positive.

Drug	Dosages and Routes	Doses	Schedule (Day)
Prednisolone ‡	40 $mg/m^2/d$ PO (tid)	84	1-28 (28 days)<br>Tapering in 1 week
Dexamethasone (for ETP immunophenotype)	10 $mg/m^2/day$ PO (divided t.i.d.)	63	Days 1-21
	4 $mg/m^2/day$ PO (divided t.i.d)	9	Days 22-24
	2 $mg/m^2/day$ PO(divided t.i.d)	12	Days 25-28
Zantac (only for induction; may not be given in infant ALL)			
Vincristine	1.5 mg/m^2 (maximum 2 mg)	4	1, 8, 15, 22
Epirubicin	20 mg/m^2	2	1, 8*
L-asparaginase	6,000 U/m^2 IM **	6	3, 5, 7, 10, 12, 14
TIT	<u>The first TIT is at the disappearance of blast from PB, no later than Day 10</u>. Subsequent TITs depend on CSF finding and risk group.		

<u>Further treatment is based on the result of MRD on day 15</u>

If Day 15, MRD < 1%

L-asparaginase		3	17, 19, 21

If Day 15, MRD ≥ 1% and < 5%

L-asparaginase		6	17, 19, 21, 24, 26, 28
Cyclophosphmide	1000mg/m^2 IV	1	22†
Cytarabine	75mg/m^2/dose IV	8	Days 23-26, 30-33†
6-mercaptopurine	60mg/m^2/dose	14	Days 22-35†

Dasatinib (*Ph*+)§	60mg/m^2/day	Daily	Starting Day 15 of induction to continue until end of treatment
**If Day 15, MRD ≥ 5%**			
L-asparaginase		6	17, 19, 21, 24, 26, 28
Cyclophosphmide	300mg/m^2 IV	4	q12 hrs on Days 22-23†
Cytarabine	75mg/m^2/dose IV	8	Days 23-26, 30-33†
6-mercaptopurine	60mg/m^2/dose	14	Days 22-35†
Dasatinib (*Ph*+)§	60mg/m^2	Daily	Starting Day 15 of induction to continue until end of treatment

* Day 8 dose may not be given or be delayed in apparent standard-risk patients with clearance of blasts and leukopenia

** No special concern of risk of bleeding; IM irrespective of platelet count.

§ May be given to cases with other genetic abnormalities such as *EBF1-PDGFRB* or *NUP214-ABL1*

‡ Oral prednisone can be substituted with methylprednisolone at 20 mg/m2/day IV (t.i.d.) for patients who cannot tolerate the oral medication

†May be delayed for 3 to 7 days if Day 22 WBC<1000 and Day 22 APC<300 and the last few doses of cytarabine and 6-mercaptopurine may be omitted if the patient develops infection with leukopenia and/or neutropenia.

-To avoid dry tap of bone marrow aspiration on day 1 and day 15, No. 16 of BMA needle is recommend.

For infant with *MLL*+

Agent	Dosage and Route	Doses	Schedule
Clofarabine	40 mg/m^2/day, 2-hour IV infusion	5	Days 22-26
Etoposide	100 mg/m^2/day, 2-hour IV infusion	5	Days 22-26
Cyclophosphamide	300 mg/m^2/day, 1-hour IV infusion	5	Days 22-26

Day 22 Vincristine will be omitted for infants with MLL+

a. No dose modifications are planned for prednisolone, asparaginase, or epirubicin therapies during induction. Only acute hemorrhagic pancreatitis or severe coagulopathy resulting in stroke syndrome warrants discontinuation of

asparaginase. In the case of mild hypersensitivity reactions (e.g., facial flushing or urticaria) to asparaginase, patients will be premedicated with diphenhydramine before the next dose. If the hypersensitivity reactions recur or there is an anaphylactic reaction, patients may switch to the Erwinia preparation (at 20,000 U/m^2 three times a week). If it is not possible to obtain Erwinia-asparaginase, no further asparaginase treatment will be administered.

b. Dose Adjustment for infants: With the exception of vincristine, all dosages given to infants (< 1 year) will be based on body surface area. For infants < 1 month of age, or for infants < 3 months of age born significantly prematurely, a 50% reduction in dosages of epirubicin, asparaginase, etoposide, methotrexate, mercaptopurine, cyclophosphamide, and cytarabine should be made. The vincristine dosage for patients < 12 months of age or < 10 kg weight is 0.05 mg/kg/dose.

c. Mild vincristine toxicity (jaw pain, constipation, decreased deep tendon reflexes) is anticipated. Only stroke-like syndrome or a motor paralysis warrants discontinuation of this drug. If persistent, severe abdominal cramps or gait impairment develop, the dose will be reduced to 1 mg/m^2. The use of azole compound (such as fluconazole, itraconazole, voriconazole), azithromycin or erythromycin may increase the toxicities of vincristine by inhibiting cytochrome P450; these drugs should be stopped one day or more before vincristine treatment.

d. On day 1, epirubicin may be delayed in patients with total bilirubin ≥ 2.0 mg/dl and direct bilirubin > 1.4 mg/dl. Omit this dose of epirubicin if total bilirubin is still ≥ 2 mg/dl and direct bilirubin > 1.4 mg/dl on day 8. Epirubicin may be given as soon as hyperbilirubinemia has resolved. The second dose of epirubicin on day 8 may be delayed in standard-risk patient who has cleared circulating blasts and has severe neutropenia, or in any risk group patient who is sick with infection. The second dose of epirubicin may be omitted in standard-risk patient if needed.

e. Trimethoprim (150 mg/m^2/day) plus sulfamethoxazole (750 mg/m^2/day) (TMP-SMZ) to prevent Pneumocystis carinii pneumonia will be given to all patients daily in two divided doses starting on day 15. After CR is achieved, TMP-SMZ will be given on Monday, Wednesday and Friday until 1 month after cessation of treatment. Adverse reactions to TMP-SMZ: For patients with rash, neutropenia, fever and other reactions presumed due to TMP-SMZ, withhold drug until reaction resolved. Do not re-challenge patients with severe exfoliative dermatitis (Stevens-Johnson syndrome), anaphylaxis or urticaria. If adverse reaction recurs, change Pneumocystis carinii

pneumonia prophylaxis to pentamidine (preferable), or atovaquone.

f. Bone Marrow Evaluations

Day 15 (or between D15 and day 19 if bone marrow procedure can not be performed on holiday) A bone marrow aspirate will be done on day 15 of remission induction to assess antileukemic response. The presence of ≥ 1% of leukemic blasts in the bone marrow by morphologic exam or by MRD study is an indication for 3 additional doses of L-asparaginase to be administered between days 24 and 28

Patients with the presence of ≥ 1% leukemic blasts in the bone marrow on day 15 receive cyclophosphamide, mercaptupurine, and cytarabine as scheduled if their clinical condition permits, regardless of their ANC. For other patients, the treatment may be delayed for 3 to 7 days to allow some degree of hematopoietic recovery if APC (ANC + monocyte) < $300/mm^3$.

End of Induction-MRD response

A bone marrow aspirate will be performed on day 38-42 of remission induction, depending on when ANC has recovered to > $300/mm^3$, WBC to > $1,000/mm^3$, and platelet count to > $50,000/ mm^3$. If the date falls on a week-end or holiday, the procedure may be performed on closest working day. MRD level will be determined in this bone marrow sample. Poor response will be defined as MRD level ≥ 0.01% (one or more lymphoblasts among 10^4 bone marrow mononuclear cells) by either immunologic or molecular assay. If the result of MRD is positive, provisional standard-risk cases will then be re-classified as high-risk (MRD ≥ 0.01% but less than 1%) or very high-risk (MRD ≥ 1%), and will receive subsequent 3 doses of HDMTX at a higher dosage (i.e., 5 gm/m^2). (These patients would have received the first HDMTX of consolidation therapy at 2.5 gm/m^2.)

g. Transfusion Guidelines

A. Bleeding is generally not a problem during induction treatment (after asparaginase treatment); therefore platelet transfusion is usually not necessary in them even though they may have thrombocytopenia, unless there is fever or mucositis.

B. There is no need to measure coagulation status during remission induction (after asparaginase treatment) because coagulopathy is expected. Unless there is bleeding complication, fibrinogen preparation or cryoprecipitate should be avoided because they can enhance the risk of thrombosis caused by asparaginase and prednisone. Note that fresh frozen plasma can supply asparagine to leukemic cells and should also be avoided.

4.2 IT Chemotherapy During Induction Treatment

As a traumatic lumbar puncture at diagnosis may result in a poorer outcome and the need for extra intrathecal therapy subsequently, all diagnostic lumbar punctures will be performed by experienced personnel, preferably under general anesthesia or deep sedation. Triple intrathecal chemotherapy (MHA) will be administered immediately after cerebrospinal fluid is collected at the disappearance of blast from PB, no later than D10. The dosage is age-dependent as following

TIT is used with dosages based on age as follows:

Age (months)	Methotrexate (mg)	Hydrocortisone (mg)	Ara-C (mg)	Volume (ml)
<12	6	12	18	6
12-23	8	16	24	8
24-35	10	20	30	10
≥ 36	12	24	36	12

Frequency and total number of triple intrathecal treatments for Remission Induction is based on the patient's risk of CNS relapse, as follows:

All patients will receive triple intrathecal treatment at the disappearance of blast from PB, no later than D10. It is suggested to perform the 2nd TIT with bone marrow evaluation on D15.

Patients with any of the following features will receive totally 4 weekly TIT during induction therapy:

- Philadelphia chromosome
- *MLL* rearrangement
- Hypodiploidy (< 44)
- WBC >100,000/mm3 at presentation
- T-cell ALL
- t (1;19)/*E2A-PBX1*
- CNS-3 status (≥ 5 WBC/μL of CSF with blasts or cranial nerve palsy)
- CNS-2 status (< 5 WBC/μL of CSF with blasts)
- Traumatic LP (> 10 RBC/μL of CSF with blasts)

Patients with any of the following features will receive TIT twice a week for 2 weeks followed by weekly TIT for 2 weeks (totally 6 TIT during induction therapy):

- T-cell ALL
- CNS-2 status (<5 WBC/μL of CSF with blasts)
- CNS-3 status (≥ 5 WBC/μL of CSF with blasts or cranial nerve palsy)
- Traumatic lumbar puncture with blasts

- t(1;19) with E2A-PBX1

Leucovorin rescue (5 mg/m^2/dose, max 5 mg) PO will be given at 24 and 30 hours after each triple intrathecal treatment during induction
Follow plasma methotrexate levels (starting 24 hours after intrathecal therapy and until level becomes undetectable) in patients with renal dysfunction or extra fluid in third space, and rescue with leucovorin.

It is also important to correct hypertension and to prevent constipation during remission induction because patients with these features are at high risk of seizure (posterior reversible encephalopathy syndrome). Avoid syndrome of inappropriate antidiuretic hormone secretion from vincristine treatment.

4.3 Consolidation Treatment (8 weeks)
When WBC >1500/mm^3, ANC >300/mm^3, platelet count >50,000/mm^3, and renal function is normal, consolidation treatment will be started.

Drug Dosages

VHR, HR	SR
MTX 5 gm/m^2 IV drip D1, 15, 29, 43	MTX 2.5 gm/m^2 IV drip D1, 15, 29, 43
6-MP 40 mg/m^2/day D1-56	6-MP 40 mg/m^2/day D1-56
TIT D1, 15, 29, 43 (8-12 hrs before HDMTX)	TIT D1, 15, 29, 43 (8-12 hrs before HDMTX)

The subsequent dose of HDMTX, 6-MP and IT will be delayed if WBC < 1,000/mm^3, ANC < 300/mm^3, platelet count < 50,000/mm^3, SGPT > 500U/L, total bilirubin > 2 mg/dl and direct bilirubin > 1.4 mg/dl, mucositis is present, or renal function is abnormal.

Pre-hydration
At least two hours before high dose methotrexate, prehydration IV fluid (D5W + 40 mEq NaHCO3/L + 20 mEq KCl/L) will be administered at the rate of 200 ml/m^2/hr. At start of prehydration, one IV dose of NaHCO3 (unless otherwise clinically indicated, 12 mEq/m^2 for standard-risk patients and 25 mEq/m^2 for high-/very high-risk patients) diluted in 50 ml D5W will be given over 15 minutes. Prehydration fluid may also be given overnight at a rate of at least 100 ml/m^2/hr, especially in patients who had delayed clearance with prior course. High dose methotrexate treatment will follow, provided that urinary pH is >6.5; exceptions must be cleared with the pharmacokinetics service

and the attending physician.

High Dose Methotrexate Infusion

Methotrexate loading dose will be given over 1 hour, followed immediately by maintenance infusion over 23 hours. During the methotrexate infusion, patients should receive hydration fluid with D5W + 40 mEq/L NaHCO3 + 20 mEq KCl/L at 100-150 ml/m^2/hr. Urine PH will be monitored with each void during infusion. An IV bolus of 12 mEq/m^2 NaHCO3 will be given if urine pH is 6.0; and 25 mEq/m^2 will be given if urine pH is <6.0. Acetazolamide 500 mg/m^2 orally every 6 to 8 hours may be used if systemic alkalosis limits the administration of bicarbonate for urinary alkalinization. Patients with evidence of renal dysfunction or delayed clearance during the methotrexate infusion may receive less than a 24 hour methotrexate infusion.

Leucovorin rescue

Leucovorin, 15 mg/m^2 (IV or PO) for high-/very high-risk or 10 mg/m^2 (PO or IV) for standard-risk cases, will be started at 42 hours after the start of methotrexate and repeated every 6 hours for a total of three doses. The dosage of leucovorin will be increased in patients with high plasma methotrexate concentrations (>1.0μM at 42 hours) and continued until the methotrexate concentration is less than 0.10μM. Additional measures, such as hydration, hemoperfusion, or carboxypeptidase will be considered in patients with 42-hour methotrexate levels > 10μM. Patients with a history of delayed Grade 3 or 4 gastrointestinal toxicity with prior methotrexate or a history of typhlitis with any chemotherapy should have leucovorin continue for 5, rather than 3 doses; those with early toxicity should have leucovorin begin at 36 hours with subsequent methotrexate; if toxicity recurs, the baseline leucovorin dosage should also be increased. Blood counts should be followed after high-dose methotrexate twice weekly; 6-MP dose should be reduced to half dose (20 mg/m^2/day) if WBC is between 1000 to 1500/mm^3, and should be held if WBC is less than 1000/mm^3.

Avoid the use of concomitant Bactrim or penicillin during high-dose methotrexate treatment because they will delay methotrexate clearance.

4.4 REINTENSIFICATION TREATMENT (for Very High-Risk ALL)

Patients with very high-risk leukemia may receive reintensification therapy and then will be offered the option of transplant. This treatment will attempt to maximize leukemic cell kill before allogeneic hematopoietic stem cell transplantation (HSCT). For patients with Philadelphia chromosome positive ALL and positive MRD at the end of induction, ETP T-ALL, and those with induction failure or >1% leukemic lymphoblasts (determined by MRD study) in bone marrow at the end of remission induction (with the exception of hyperdiploid >50 and *TEL-AML1* cases who should have positive MRD after consolidation therapy), treatment will be given after consolidation therapy. However, consolidation therapy may be shortened, depending on patient's response to therapy and on the timing of transplantation. For patients with > 0.1% leukemic lymphoblasts (determined by MRD study) in bone marrow in week 7 of continuation treatment, this treatment will be given after the reinduction I. Upon marrow recovery (i.e., ANC≥300/mm^3, WBC≥1000/mm^3 and platelet count≥50,000/mm^3) after each course of reintensification, bone marrow examination with MRD study will be repeated.

This treatment course may be repeated only once if the patient still has persistently positive MRD (i.e. ≥ 0.01% blasts). Allogeneic hematopoietic stem cell transplantation may proceed after 1 course of the treatment if MRD becomes negative with the first course of treatment; otherwise, transplant will be performed after two courses of treatment. Patients deemed unsuitable for the transplant or who decline the procedure or whose donor has yet to be identified, will remain on study and receive subsequent chemotherapy as scheduled. The treatment scheme and dosage of chemotherapy are summarized below.

Agent	Dosage and Route	Doses	Schedule
Dexamethasone	20 mg/m^2/day PO or IV (divided t.i.d)	18	Days 1-6
Cytarabine	2 grams/m^2, 3-hour IV infusion every 12 hours	4	Days 1-2
Etoposide	100 mg/m^2, 1-hour IV infusion every 12 hours	5	Days 3-5
TIT		1	Day 5
L-asparaginase	25,000iu/m^2 IM	1	Day 6

Patients with suboptimal response to reintensification may receive one to two cycles of clofarabine/cyclophosphamide/etoposide/dexamethasone:

Agent	Dosage and Route	Doses	Schedule
Clofarabine	40 $mg/m^2/day$, 2-hour IV infusion	5	Days 1-5
Etoposide	100 $mg/m^2/day$, 2-hour IV infusion	5	Days 1-5
Cyclophosphamide	300 $mg/m^2/day$, 30-60 minute IV infusion	5	Days 1-5
Dexamethasone	8 $mg/m^2/day$ (divided t.i.d)	15	Days 1-5

4.5 Continuation Treatment (120 weeks)

Post-remission continuation treatment begins after the completion of consolidation, provided that the ANC> 300/mm^3, WBC >1500/mm^3 and platelet count >50,000/mm^3 as well as no evidence of mucositis. Continuation treatment (120 weeks) differs according to the risk classification, as follows (abbreviations as defined below).

Treatment (Weeks 1 to 20)

Week	VHR/HR	SR
1	DEX + EPI + VCR + 6MP + ASP§	6MP + DEX + VCR
2	6MP + ASP	6MP + MTX
3	#6MP + ASP	*6MP + MTX
4	DEX + EPI + VCR + 6MP + ASP	6MP + DEX + VCR
5	6MP + ASP	6MP + MTX
6	6MP + ASP	6MP + MTX
7	* †Reinduction I	*Reinduction
8	Reinduction I	Reinduction
9	Reinduction I	Reinduction
10	6MP + ASP	6MP + MTX
11	EPI + VCR + 6MP + ASP	6MP + MTX
12	*6MP + ASP	* 6MP + MTX
13	6MP + ASP	6MP + MTX
14	DEX + EPI + VCR + 6MP + ASP	6MP + DEX + VCR
15	6MP + ASP	6MP + MTX
16	6MP + ASP	6MP+ MTX
17	* †Reinduction II	*6MP + DEX + VCR
18	Reinduction II	6MP + MTX
19	Reinduction II	6MP + MTX
20	6MP + MTX	6MP + DEX + VCR

§ First dose of ASP given at day 3 (after 2 days of DEX) to reduce the risk of ASP allergy.

#Triple intrathecal treatment will be given to other high-risk/very high-risk cases with WBC ≥100,000/mm^3, T-cell ALL with WBC ≥ 50,000/mm^3, presence of Philadelphia chromosome, *MLL* rearrangement, hypodiploidy <44, or CNS-3 status, with CNS-2 or traumatic lumbar puncture with blasts at diagnosis..

*IT MHA (methotrexate + hydrocortisone + cytarabine)

† MRD study before each reinduction therapy and at the end of therapy will be done in patients with positive MRD at end of remission induction. Bone marrow sample will be used in B-lineage All and blood sample can be used for T-lineage ALL.

Patients with MRD ≥ 0.1% at week 7 receive reintensification treatment after Reinduction I

Dexamethasone, vincristine and L-asparaginase can be given regardless of blood counts,
provided that the patient is not sick. Methotrexate, mercaptopurine and epirubicin will be held if ANC <300/mm^3, APC<500/mm^3, WBC <1,000/mm^3, or platelet count <50,000/mm^3.
(‡) Continue Dasatinib in cases with Ph, EBF1-PDGFRB or NUP214-ABL1

Drug dosages, schedules and routes for continuation therapy weeks 1 to 6 and 10 to 16

DEX (dexamethasone)	12 mg/m^2(VHR/HR) or 8 mg/m^2 (SR) PO daily (tid) x 5 days, Days 1-5
EPI (epirubicin)	30 mg/m^2 IV, Day 1
VCR (vincristine)	2.0 mg/m^2 IV push (max. 2 mg), Day 1 (0.05 mg/kg for patients < 1 year of age or < 10kg in weight)
6MP (6-mercaptopurine)	40 mg/m^2 PO h.s.daily x 7 days (VHR/ HR), Days 1-7<br>50 mg/m^2 PO h.s. daily x 7 days (SR), Days 1-7
ASP (L-asparaginase)	10,000 U/m^2 IM, Day 1
MTX (methotrexate)	40 mg/m^2 IV or IM, Day 1

REINDUCTION TREATMENT
This phase of treatment will be started at weeks 7 and/or 17 if patients have ANC > 500/mm^3, WBC >1500/mm^3, and platelet count > 50,000/mm^3.

Intrathecal treatment will be followed by leucovorin rescue (5 mg/m^2/dose PO, max 5 mg) at 24 and 30 hours **<u>only in patients with prior CNS toxicities or in patients with WBC < 1500/mm3, or ANC < 500/mm^3</u>**

Reinduction I for VHR/HR ALL excluding infant with *MLL*+(3 weeks)

Agents	Dosages and routes	# Doses	Schedules
Dexamethasone	12 mg/m^2/day PO (t.i.d.)	45	Days 1-8, 15-21
Vincristine	1.5 mg/m^2/week IV (max 2 mg)	3	Days 1, 8, 15
Epirubicin	30 mg/m^2	2	Days 1,8
L-asparaginase	6,000 U/m^2/thrice weekly IM	9	Days 3, 5, 7, 10, 12,

			14,17,19,21
Methotrexate + hydrocortisone + ara-C	Age-dependent, IT	1	Day 1

Reinduction II for VHR/HR ALL including infant with *MLL*+ (3 weeks)

Agents	Dosages and routes	# Doses	Schedules
Dexamethasone	12 mg/m²/day PO (t.i.d.)	45	Days 1-8, 15-21
Vincristine	1.5 mg/m²/week IV (max 2 mg)	3	Days 1, 8, 15
L-asparaginase	6,000 U/m²/thrice weekly IM	9	Days 3, 5, 7, 10, 12, 14,17,19,21
Methotrexate + hydrocortisone + ara-C	Age-dependent, IT	1	Day 1

Reinduction for SR ALL (3 weeks)

Agents	Dosages and routes	Doses	Schedules
Dexamethasone	10 mg/m²/day PO (t.i.d.)	45	Days 1-8, 15-21
Vincristine	1.5 mg/m²/week IV (max 2 mg)	3	Days 1, 8, 15
L-asparaginase	6,000 U/m² thrice weekly IM	9	Days 3, 5, 7, 10, 12, 14,17,19,21
Epirubicin	30 mg/m²/week IV	1	Day 1
Methotrexate + hydrocortisone + ara-C	Age-dependent, IT	1	Day 1

Reinduction I for infant with *MLL*+

Agent	Dosage and Route	Doses	Schedule
Dexamethasone	8 mg/m²/day (divided t.i.d)	45	Days 1-8; Days 15-21
Clofarabine	40 mg/m²/day, 2-hour IV infusion	5	Days 1-5
Etoposide	100 mg/m²/day, 2-hour IV infusion	5	Days 1-5
Cyclophosphamide	300 mg/m²/day, 1-hour IV infusion	5	Days 1-5

L-asparaginase	6,000 U/m² thrice weekly IM	9	Days 3, 5, 7, 10, 12, 14, 17, 19, 21
Methotrexate + hydrocortisone + ara-C	Age-dependent, IT	1	Day 1

For infants < 1 month of age, or for infants < 3 months of age born significantly prematurely, a 50% reduction in dosages of asparaginase, etoposide, cyclophosphamide, epirubicin, and clofarabine should be made.

Treatment (weeks 21 to end of therapy)

Week	HR	SR
21	6MP + MTX	6MP + MTX
22	6MP + MTX	6MP + MTX
23	Cyclo + Ara-C	6MP + MTX
24	*DEX + VCR	*6MP + DEX + VCR
25	6MP + MTX	6MP + MTX
26	6MP + MTX	6MP + MTX
27	Cyclo + Ara-C	6MP + MTX
28	*DEX + VCR	(*)6MP + DEX + VCR

***TIT**

(*)IT MHA for low-risk cases with WBC > 100,000/mm³ , CNS-2 or traumatic lumbar puncture with blast.

Drug Dosages, Schedules and Routes for Continuation Therapy from Week 21 to End of Therapy

6MP (6-mercaptopurine)	60 mg/m² PO h.s. daily x 7 days, Days 1-7
MTX (methotrexate)	40 mg/m² IV or IM(or PO, if parenteral route is not feasible), Day 1
Cyclo (Cyclophosphamide)	300 mg/m² IV, Day 1 (VHR/HR)
Ara-C (Cytarabine)	300 mg/m² IV, Day 1 (VHR/HR)
DEX (dexamethasone)	12 mg/m² (VHR/HR) or 8 mg/m² (SR) PO daily (tid) x 5, Day 1-5
VCR (vincristine)	2.0 mg/m² IV push (max. 2 mg), Day 1

The same treatment (weeks 21-28) will be repeated for a total of 6 times (until week 68). After week 68, cyclophosphamide and cytarabine will be replaced by daily 6MP and methotrexate; all patients will then receive daily 6MP and weekly MTX with pulses of dexamethasone and vincristine every 4 weeks until week 100, after which only 6MP and methotrexate will be given.

Cyclophosphamide and cytarabine dosages may need to be reduced by 33% to 50% in

patients who repeatedly have very low counts (WBC<1000/mm^3 or ANC<300/mm^3 or platelets <50,000/mm^3) one to two weeks later.

Dexamethasone dose decreases to 6 mg/m^2 beginning week 68.

Intrathecal treatment will be given every 8 weeks only to patients at high risk of CNS relapse after week 48 and will be discontinued after week 96. Continuation therapy will be discontinued after 120 weeks.

4.6 IT Chemotherapy During Continuation Treatment

-Triple intrathecal treatment will be given to SR cases with CNS-1 status (no identifiable blasts in CSF) on weeks 3, 7, 12, 17, 24, 32, 40, and 48. (14 times)
-Triple intrathecal treatment will be given to SR cases with CNS-2 or traumatic CSF with blasts status on weeks 3, 7, 12, 17, 24, 28, 32, 36, 40, 44 and 48. (19 times)
-Triple intrathecal treatment will be given to HR cases with CNS-1 status on weeks 3, 7, 12, 17, 24, 28, 32, 36, 40, 44 and 48. (17 Times)
-Triple intrathecal treatment will be given to other high/very high-risk cases with WBC≥100,000/mm^3 at presentation, , T-cell ALL, t (1;19)/*E2A-PBX1,*presence of Philadelphia chromosome, *MLL* rearrangement, hypodiploidy <44, CNS-2 or CNS-3 status, or traumatic lumbar puncture with blasts on weeks 3, 7, 12, 17, 24, 28, 32, 36, 40, 44, 48, 56, 64, 72, 80, 88 and 96. (25 Times)

Leucovorin will not be given after intrathecal treatment during continuation treatment unless the patient has an adverse reaction with previous intrathecal or methotrexate treatment, e.g., seizure or encephalopathy, has renal dysfunction resulting in high plasma methotrexate concentration, or has Down syndrome. Leucovorin may be given when patient is neutropenic, at treating physician's discretion; however, it is generally preferable to delay intrathecal therapy if patient has neutropenia. Down syndrome patients will receive leucovorin with every LPIT.

Note that WBC and ANC counts should be double a week following dexamethasone pulse therapy. If WBC or ANC counts fail to double (indicating low bone marrow reserve), 6-MP and MTX dosages should be reduced to half. If WBC or ANC remains the same or is lower, 6-MP and MTX should be held because the patient is at high risk of infection, and blood counts should be repeated in 3 to 4 days to decide if 6-MP can be resumed. Patients 10 years of age or old are at especially high risk of sepsis. If patient has abdominal pain, typhlitis must be excluded and antibiotics may be started even if the patient has no fever.

5. DRUG INFORMATION

Prednisolone

As a synthetic adrenal steroid, prednisolone binds with steroid receptors on the nuclear membrane to impair cellular mitosis and inhibit protein synthesis. Catabolism is hepatic with excretion in the urine. Biologic half-life is 12-30 hours. This drug is available in 5.0 mg tablets or 1.0 mg/ml solution for oral administration. Toxicity includes fluid retention, hypertension, increased appetite, transient diabetes, acne, striae, personality changes, Cushingoid syndrome, possible peptic ulcer and immunosuppression. The excitement can be treated by potassium supplement. Long-term therapy is associated with osteoporosis and growth retardation.

Vincristine

As a sulfate salt derived from periwinkle plant, it is a tubulin binder, which inhibits mitosis and arrests cell division in metaphase. It is excreted in the bile and feces with a triphasic (0.85 – 164 minutes) plasma half-life. This drug is commercially available in 1 mg vials; it is light sensitive and should be refrigerated. VCR is given IV. Toxicity includes: peripheral neuropathy manifested by constipation, ileus, ptosis, vocal cord paralysis, jaw pain, abdominal pain, weakness on extremities and loss of deep tendon reflexes. The neuropathy can be treated with gabapentin (Neurotin) and/or vitamin B6. Other adverse reactions include immunosuppression, alopecia, local ulceration with extravasation and a syndrome of inappropriate excretion of ADH (SIADH). Liver dysfunction may enhance toxicity. Azols, grapefruit, phenobarbitol and Dilantin will change its efficacy. Special precautions to avoid extravasation should be used. The maximal dose of VCR for all patients on this study is 2.0 mg IV.

Epirubicin (4′ – epidoxorubicin)

It is a derivative of doxorubicin obtained by epimerization of the hydroxyl group in position 4′ in the sugar moiety, with antineoplastic actions similar to those of doxorubicin and **less cardiotoxicity** as compared to that of doxorubicin. It is available in 10 mg vials. Following intravenous administration epirubicin is rapidly and extensively distributed into body tissues, and undergoes metabolism in the liver. It is eliminated mainly in bile, with a terminal plasma elimination half-life of about 40 hours. Toxicity is increased by hepatic dysfunction. The toxicities include myelosuppression, nausea, vomiting, mucositis, alopecia, and severe ulceration if extravasation occurs. Special precautions to avoid extravasation are used.

L-Asparaginase

Extracts of E. coli L-asparaginase impair asparagine synthesis and are lethal in all phases of the cell cycle to cells that cannot synthesize the essential amino acid asparagine. L-asparaginase is a potent inhibitor of cellular protein synthesis. Plasma half-life varies from 3 – 30 hours with detectable blood levels of 13 – 22 days. Excretion is through bile and urine. E. coli L-asparaginase is available at 5,000 U/vial. These drugs are given by deep IM injection.

Toxicities include hypersensitivity reactions with local rash, urticaria or full anaphylaxis (laryngeal stridor, hypotension, shock). Other toxicities include transient diabetes, elevation of blood ammonia, and abnormalities of blood clotting screens due to decreased coagulation factor synthesis. Rarely, bleeding may occur secondary to asparaginase-induced decrease of liver-synthesized clotting proteins. Thrombosis can also occur due to rebound of clotting protein synthesis. A rare toxicity that may require discontinuation of this drug is acute pancreatitis. Other complications include: immunosuppression, malaise, anorexia, abnormal liver function tests (common), somnolence, and lethargy. Administration is IM on this protocol. Because of the possibility of an anaphylactic reaction, patients must be observed for at least one hour in a setting where resuscitation equipment is available. IM administration lessens the risk of anaphylactic and allergic reactions compared to IV administration. What we use is from Kyowa Hacco Kogyo, Japan, which has twice pharmacologic potency as compared to other products (TPOG: Leukemia 13:155-160, 1999)

If allergy occurs, replace Leunase with Erwinase 20000U/m^2

Cytosine arabinoside (cytarabine, Ara-C)

As a synthetic analogue of the nucleoside deoxycytidine, it undergoes phosphorylation to ara-CTP, a competitive inhibitor of DNA polymerase. This drug is a cell cycle specific S-phase inhibitor of DNA synthesis. Initial plasma half life is 15 minutes, with a secondary phase of 2 hours. This drug is available in 100 and 500 mg/vial. The drug is stable for at least 2 years at room temperature. The diluent contains 0.9% benzyl alcohol in water. The reconstituted solution should be stored at room temperature and used within 48 hours. Toxicity consists of severe leukopenia and thrombocytopenia. Other adverse reactions include immunosuppression, nausea, vomiting, anorexia, stomatitis, gastrointestinal ulceration, flu-like syndrome with fever, alopecia and skin rash. Diarrhea, fever, somnolence, conjunctivitis, ataxia, cerebellar neuropathy, or veno-occlusive disease can also develop. IT administration can result in fever, headache, vomiting and pleocytosis. On rare occasions meningismus, convulsions, paresis and cardiac arrest have been reported.

In this study, Ara-C will be administered IV over 1 hour or IV push (continuation).

Anti-emetics are indicated with IV administration. For intrathecal administration, preservative-free Ara-C is used.

Methotrexate (MTX)

This folate analogue which competitively inhibits dihydrofolate reductase resulting in inhibition of DNA, RNA and protein synthesis. IV half life is initially 1.2 hours, with a secondary phase of 10.4 hours. For high dose MTX infusions, vials each containing either 500 mg or 1000 mg of MTX without preservatives are available. For intrathecal therapy, MTX is available as a 20 mg or 50 mg preservative-free vial. Acute toxicity includes myelosuppression (nadir 7-14 days), ulcerative stomatitis, and diarrhea. With high doses, other adverse sequelae include glossitis, stomatitis, alopecia, hepatic toxicity, impaired renal function and malaise. Late effects infrequently reported include pulmonary dysfunction and osteoporosis. Intrathecal administration has been associated with headache, arachnoiditis, convulsion, and paresis. **When given in high systemic doses in combination or after cranial irradiation, a syndrome of progressive CNS encephalopathy occasionally leading to death has been reported. Because of the potential of the adverse pharmacologic interaction, patients should not receive nonsteroidal anti-inflammatory drugs concomitantly or within 1 week of receiving HDMTX.

For high dose therapy, rescue with calcium leucovorin and vigorous hydration is mandatory.

6-Mercaptopurine (6-MP)

As a synthetic purine analogue, it is metabolized to an active ribonucleoside form for incorportion into DNA as a false base. Other metabolic effects are inhibition of de novo purine synthesis. This drug has a 90 minute plasma half life and rapid renal excretion. Oral aborption is erratic. 6-MP is available as 50 mg oral tablets which are stable at room temperature when protected from light. Toxicities include liver function abnormalities, rash, anorexia, and leukopenia. Leukopenia is mild in standard dosage with the nadir of counts 10-14 days from dosage. Allopurinol, a xanthine oxidase inhibitor used for treatment of hyperuricemia, enhances toxicity of this drug. 6-MP will be given orally on this protocol at bedtime, 1.5 hours before meal or 2 hours after meal.

Leucovorin (citrovorum factor)

An "activated" tetrahydrofolate, leucovorin bypasses the inhibition of dihydrofolate reductase by MTX and is used as an "antidote" to high doses of MTX to protect against undue methotrexate toxicity. The drug is 90% absorbed orally at doses <50 mg, has a

serum half life of 35-45 minutes, and is excreted by the kidneys. Leucovorin is available in 5 and 15 mg tablets and in 3, 15 and 50 mg vials for IV use. Toxicity is rare with allergic sensitization after oral or IV use having been reported.

Cyclophosphamide (Cytoxan, Endoxan)

Cyclophosphamide, an alkylating agent, inhibits DNA replication by binding to DNA strands. Cyclophosphamide will be given IV in this study. Toxicity includes alopecia, nausea, vomiting, stomatitis, diarrhea, myelosuppression, immunosuppression, hemorrhagic cystitis and inappropriate secretion of ADH. Cardiac necrosis has been reported with single doses in excess of 200 mg/kg.

Dexamethasone

It is a glucocorticoid and has the similar toxicity described under prednisolone. However, it has little or no effect on sodium and water retention. Dexamethasone is readily absorbed from the gastro-intestinal tract. Its biological half-life in plasma is about 190 minutes. Binding of dexamethasone to plasma proteins is less than for most other corticosteroids. This drug is available in 0.5 mg tablets for oral administration.

Trimethoprim-sulfamethoxazole (TMP-SMZ)

TMP-SMZ is a combination of two antimicrobial drugs that provides broad-spectrum antibacterial and anti-Pneumocystis carinii effects. TMP binds dihydrofolate reductase and SMZ competes for paraaminobenzoic acid in folate synthesis of microbes. Toxicity: rash, agranulocytosis, neutropenia, nausea, vomiting and anorexia. Discontinue after the cessation of chemotherapy, for 1 month.

ETOPOSIDE (VP-16) (VepesidÒ)

Source and Pharmacology: Etoposide is an epipodophyllotoxin derived from *Podophyllum pelatatum.* It is thought to act mainly by inhibiting topoisomerase II, causing double and single strand DNA breaks. Etoposide is cell cycle, phase-specific, with activity in the G2 and S phases. Absorption of etoposide is approximately 30-40% and is highly variable and somewhat dosedependent. It is extensively bound to serum proteins and is metabolized in the liver, including cytochrome P450 3A metabolism to several moieties that include a reactive oxidized species. Etoposide and its metabolites are excreted mainly in the urine with a smaller amount excreted in the feces. Dosage adjustments should be considered in patients with liver dysfunction, kidney dysfunction or hypoalbuminemia.

Formulation and Stability: Etoposide is available in multi-dose vials containing 100mg

of etoposide as a 20mg/ml solution and 30% alcohol. Etoposide is also available as a 50 mg capsule. The intact vials of etoposide solution should be stored at room temperature. The capsules should be stored under refrigeration. Etoposide solution should be diluted in D5W or 0.9% NaCl prior to administration. Solutions with a final concentration of 0.2 and 0.4 mg/ml are stable at room temperature for 96 hours and 24 hours respectively. Toxicity: Dose limiting toxicity is myelosuppression. Nausea and vomiting (usually of low to moderate severity), diarrhea, mucositis (particularly with high doses), alopecia and anorexia are fairly common. Hypotension can occur with rapid infusions. Other side effects reported less commonly include hepatitis, fever and chills, anaphylaxis and peripheral neuropathy. Secondary leukemia has been reported.

CLOFARABINE (Cl-F-Ara-A, CAFdA, 2-Chloro-9-(2-deoxy-2-fluoro-beta-Darabinofuranosyl)-9H-purin-6-amine, Clofarex, Clolar™)

Source and Pharmacology: Clofarabine is sequentially metabolized intracellularly to the 5′-monophosphate metabolite by deoxycytidine kinase and mono- and di-phosphokinases to the active 5′-triphosphate metabolite. Clofarabine has high affinity for the activating phosphorylating enzyme, deoxycytidine kinase, equal to or greater than that of the natural substrate, deoxycytidine. Clofarabine inhibits DNA synthesis by decreasing cellular deoxynucleotide triphosphate pools through an inhibitory action on ribonucleotide reductase, and by terminating DNA chain elongation and inhibiting repair through incorporation into the DNA chain by competitive inhibition of DNA polymerases. The affinity of clofarabine triphosphate for these enzymes is similar to or greater than that of deoxyadenosine triphosphate. In preclinical models, clofarabine has demonstrated the ability to inhibit DNA repair by incorporation into the DNA chain during the repair process. Clofarabine 5′-triphosphate also disrupts the integrity of mitochondrial membrane, leading to the release of the pro-apoptotic mitochondrial proteins, cytochrome C and apoptosis-inducing factor, leading to programmed cell death. Clofarabine is cytotoxic to rapidly proliferating and quiescent cancer cell types in vitro.

The population pharmacokinetics of clofarabine was studied in 40 pediatric patients aged 2 to 19 years (21 males/19 females) with relapsed or refractory ALL or AML. At the given 52 mg/m^2 dose, similar concentrations were obtained over a wide range of BSAs. Clofarabine was 47% bound to plasma proteins, predominantly to albumin. Based on non-compartmental analysis, systemic clearance and volume of half-life was estimated to be 5.2 hours. No apparent difference in pharmacokinetics was observed between patients with ALL and AML or between males and females. Based on 24-hour urine collections in the pediatric studies, 49-60% of the dose is excreted in the urine

unchanged. *In vitro* studies using isolated human hepatocytes indicate very limited metabolism (0.2%), therefore the pathways of non-renal elimination remain unknown. Although no clinical drug-drug interaction studies have been conducted to date, on the basis of the *in vitro* studies, cytochrome p450 inhibitors and inducers are unlikely to affect the metabolism of clofarabine. The effect of clofarabine on the metabolism of cytochrome p450 substrates has not been studied. The pharmacokinetics of clofarabine has not been evaluated in patients with renal or hepatic dysfunction.

Formulation and Stability: Clofarabine (1 mg/mL) is supplied in a 20 mL, single-use vial. The 20 mL vial contains 20 mg clofarabine formulated in 20 mL unbuffered normal saline (comprised of Water for Injection, USP, and Sodium Chloride USP). The pH range of the solution is 4.5 to 7.5. Store at 25°C (77°F); excursions permitted to 15-30°C (59-86°F).

Toxicity: The most common toxicities of clofarabine are vomiting, nausea, diarrhea, anemia, leukopenia, thrombocytopenia, neutropenia, febrile neutropenia, and infection. Greater than 10% of patients receiving clofarabine have the following adverse events: tachycardia, abdominal pain, constipation, gingival bleeding, sore throat, edema, fatigue, injection site pain, lethargy, mucosal inflammation, pain, pyrexia, rigors, hepatomegaly, jaundice, weight loss, anorexia, arthralgia, myalgia, back pain, limb pain, dizziness, headache, somnolence, tremor, anxiety, depression, irritability, hematuria, cough, dyspnea, epistaxis, pleural effusion. Respiratory distress, confusion, dermatitis, dry skin, erythema, palmar-plantar erythrodysesthesia syndrome, petechiae, pruritus, flushing, hypertension, hypotension, increases in ALT, AST, and bilirubin, transient left ventricular systolic dysfunction, and increased serum creatinine. Four of 113 pediatric patients experienced capillary leak syndrome or SIRS leading to multi-organ failure. Fetal and teratogenic effects have been noted in animals. It is not known whether clofarabine or its metabolites are excreted in human milk.
Guidelines for Administration: See Treatment and Dose Modification sections of the protocol. Filter clofarabine through a sterile 0.2 μm syringe filter and then further dilute with 5% dextrose injection USP or 0.9% sodium chloride injection USP to a convenient volume and infuse over 2 hours. The resulting admixture may be stored at room temperature, but must be used within 24 hours of preparation.

To reduce the effects of tumor lysis and other adverse events it is recommended that continuous IV fluids be given throughout the 5 days of clofarabine administration. Since clofarabine is primarily excreted through the kidneys, drugs with known renal toxicity should be avoided during the 5 days of clofarabine administration. In addition,

since the liver is a known target organ for clofarabine toxicity, concomitant use of medications known to induce hepatic toxicity should be avoided.

DASATINIB (SPRYCEL)

FOR PH+ ALL PATIENTS ONLY

Source and Pharmacology: Dasatinib (an aminothiazole analogue) is an inhibitor of multiple tyrosine kinases. It is approved for the treatment of chronic myelogenous leukemia (CML) and for the treatment of adults with Philadelphia chromosome-positive ALL with resistance or intolerance to prior therapy. It is being investigated as a broad-spectrum antitumor agent against solid tumors. Dasatinib is a potent, broad spectrum ATP-competitive inhibitor of 5 critical oncogenic tyrosine kinase families: BCR-ABL, SRC family kinases, c-KIT, ephrin (EP) receptor kinases, and PDGF receptor. Each of these protein kinases has been strongly linked to multiple forms of human malignancies. In adults, the maximum plasma concentration of dasatinib after oral administration is observed between 0.5 and 6 hours. The observed effects from food were not clinically relevant. The overall terminal half-life is 3-5 hours. The drug exhibits dose proportional increases in AUC and linear elimination characteristics over the dose range of 15 mg to 240 mg/day in adults. Dasatinib is primarily metabolized in the liver by the human CYP3A4 enzyme, is a significant inhibitor of CYP3A4. Dasatinib may decrease the metabolic clearance of drugs that are significantly metabolized by the CYP3A4 enzyme. Due to the potential of dasatinib to prolong the QT/QTc, caution must be used when administering it with other potential QTc prolonging medications. Due to the possibility of gastrointestinal, cardiac, and cutaneous hemorrhage, avoid using medications that inhibit platelet function or anticoagulants in conjunction with Dasatinib. Dasatinib is not a p - glycoprotein inhibitor.

Formulation and Stability: Dasatinib is available as tablets of 20 mg, 50 mg, or 70 mg. The core tablet is surrounded by a film coating to prevent exposure to the active drug substance during handling. If tablets are cut or crushed, procedures to prevent exposure to the active drug substance should be followed. Pregnant women or breastfeeding mothers should not handle crushed and/or broken dasatinib tablets.

The intact dasatinib tablets can be placed (and allowed to dissolve) in 1 ounce of lemonade (a double strength juice is recommended to obscure the bitter taste), or 1 ounce of preservative-free apple juice, or 1 ounce of preservative-free orange juice. The following (i.e. steps 1 thru 7) is the procedure for the preparation of the lemonade dosing solution. For preservative-free apple juice or preservative-free orange juice, steps 2 thru 7 should be followed.

Mix the contents of one 12 ounce can of Minute Maid Premium Frozen Concentrate with 2 cans (i.e. the emptied lemonade container) of water. This will produce lemonade that is a little more than twice as concentrated as the instructions on the can with a sweeter taste. Please keep the lemonade solution refrigerated when not in use.

Place 1 ounce (30 mL) of this lemonade solution into a drinking glass.
Place the proper dose of intact tablets into the lemonade. Please be sure to always wear protective gloves when handling the medication. A mask is not required when handling the medication. Always use the 1 oz of lemonade. Do not increase the lemonade volume.

Start timing for 20 minutes. At approximately the 5 minute mark, swirl the contents of the glass well for about 3 seconds.
At approximately the 15 minute mark, swirl the contents of the glass a second time. At the 20 minute mark, swirl the contents of the glass one last time. Immediately administer the entire contents of the glass.

In order to ensure administration of the entire medication dose, a rinsing step is necessary. Add 0.5 ounce (15 mL) of lemonade into the same glass that has just been emptied. Swirl the contents to remove any remaining signs of tablets from the sides or bottom of the glass. Administer the washing lemonade to the patient.
The intact bottles should be stored at controlled room temperature (15° - 25°C , 59° - 77°F) and protected from light.

Toxicity: The most frequently reported adverse events include fluid retention (pleural effusion), diarrhea, nausea, abdominal pain, rash, headache, fatigue, vomiting, bleeding events and myelosuppression. The most frequently reported serious adverse events include pyrexia, febrile neutropenia, gastrointestinal bleeding, pneumonia, thrombocytopenia, dyspnea, anemia and cardiac failure (3%). Less commonly reported are anorexia, dehydration, abdominal distension and flatulence, flushing, pruritis, elevated creatinine, and neuropathy. Elevations in transaminases and bilirubin can usually be managed with dose reductions or dose interruption. Hypocalcemia during dasatinib therapy can be managed with oral calcium supplementation.

IMATINIB (Glivec, Gleevec, Imatinib Mesylate, formerly known as STI571)
For Ph+ ALL patients only. See package insert for additional information
Source and Pharmacology: imatinib mesylate is a phenylaminopyrimidine derivative

and is a
4-[(4-Methyl-1-piperazinyl)methyl-N-[4-methyl-3-[[4-(3-pyridinyl)-2-pyrimid-inyl]amino]-phenyl]benzamidemethanesulfonate. It is a protein-tyrosine kinase inhibitor that inhibits the Bcr-Abl tyrosine kinase, the constitutive abnormal tyrosine kinase created by the Philadelphia hromosome abnormality.

Formulation and Stability: Each film-coated tablet contains 100 mg or 400 mg of imatinib freebase. The drug should be stored at 25°C (77°F); excursions permitted to 15°C-30°C (59°F -86°F). The tablets should be dispensed in a tight container, USP, and protected from moisture. The prescribed dose should be administered orally, with a meal and a large glass of water. In children, the daily dose may be split into two – once in the morning and once in the evening. For patients unable to swallow the film-coated tablets, the tablets may be dispersed in a glass of water or apple juice. The required number of tablets should be placed in the appropriate volume of beverage (approximately 50 mL for a 100-mg tablet, and 200 mL for a 400-mg tablet) and stirred with a spoon. The suspension should be administered immediately after complete disintegration of the tablet.

Toxicity: Common toxicities include dyspepsia/heartburn, nausea/vomiting, headache, myelosuppression, and fatigue. Occasional toxicities include fever, edema in limbs, face, periorbital area, weight gain, increased SGOT/SGPT, alkaline phosphatase, bilirubin, abdominal pain and cramping, myalgia, arthralgia, decreased bone marrow cellularity, lymphopenia, eczema dermatitis, rash, muscle pain and cramping, anorexia, and pigmentation changes (hypo-vitiligo). Rare toxicities include cerebral edema, melena/GI bleeding, anemia, diarrhea, dysphagia, esophagitis, odynophagia, hemorrhage/bleeding without grade 3 or 4 thrombocytopenia, pneumonitis/pulmonary infiltrates, late hepatotoxicity and decrease in the heart’s ability to pump blood.

6.TREATMENT MODIFICATIONS

Down Syndrome

Participants with Down syndrome are eligible for enrollment, with the following modifications.

A. HDMTX: Dosages of high-dose MTX will be modified due to their well documented altered pharmacokinetics and enhanced tissue sensitivity to MTX's effects. Their hydration and alkalinization regimen should be the same. However, the dose of HDMTX is 500 mg/m^2 (50 mg/ m^2 over 1 hour and 450 mg/ m^2 given over 23 hours). The baseline leucovorin rescue will begin early (at hour 30 at 30 mg/ m^2 IV q 6 hours x 2 doses, followed by 10 mg/ m^2 IV q6 hours x 6 doses). If MTX plasma levels are elevated, increased leucovorin rescue will be recommended by the Pharmaceutical Department. Vigorous hydration should be assured until the 42 hour MTX level is known.
B. Continuation low-dose MTX: The low-dose weekly MTX (40 mg/ m^2) dosage should be administered at full dosage if possible. If the patient has severe neutropenia or leucopenia (which delays subsequent therapy) or grade 4 mucositis (or mucositis which delays subsequent therapy) following the dose of 40 mg/ m^2, the dosage should be decreased to 30 mg/ m^2. If that dosage is
similarly not tolerated, then the dosage may be further decreased to 20 mg/ m^2 and finally to 10 mg/ m^2, if necessary. If 10 mg/ m^2 is also not tolerated, then leucovorin should be added at 5 mg/ m^2 every 6 hour for 4 doses starting 42 hours from the MTX dosage, with titration to acceptable toxicity.
C. Intrathecal Therapy: Intrathecal treatment should be administered as outlined in Section 4.2.
D. Down syndrome patients should be closely monitored. Dose reduction (30% to 50%) should be applied as clinically indicated (specially to dexamethasone and high-dose cytarabine for the few patients who would be treated on the standard arm, and who are noted to have higher than expected toxicity in earlier phases)

Renal Dysfunction

Subclinical renal impairment (normal serum creatinine but decreased GFR) may be present in patients receiving concurrent nephrotoxic drugs (e.g. IV acyclovir) which, if possible, should be held during and for 20 hours after HDMTX infusions or until adequate MTX clearance has been documented. Consideration to delaying MTX should be given if a patient's serum creatinine indicates renal impairment.

Hepatic Dysfunction

Anthracyclines and vincristine dosages should be modified in patients with elevated direct bilirubin concentrations or other evidence of biliary obstruction. (More conservative criteria will be used for anthracycline treatment during initial remission induction, See Section 4.1.d)
Direct bilirubin 2-4 mg/dl: 50% dosage decrease
Direct bilirubin 4-6 mg/dl: 75% dosage decrease
Direct bilirubin >6 mg/dl: withhold dose

L-asparaginase may need to be withheld in patients with elevated direct bilirubin concentrations, especially if there is evidence of mucositis.

HDMTX should be withheld if there is evidence of existing mucositis or if total bilirubin >2 mg/dl and direct bilirubin >1.4 mg/dl.

Subclinical hypertransaminasemia (SGPT >500 IU/L) is an indication to delay only high dose methotrexate but not other chemotherapy.

Obesity
Actual body weight will be used to calculate body surface area in all patients and used for dosage calculations (with the exception that vincristine dosage is capped at 2.0 mg).

Testicular Leukemia at Diagnosis
Overt testicular leukemia occurs in 2% of boys at diagnosis, generally in infants or adolescents with hyperleukocytosis. Ultrasonogram should be performed to differentiate testicular leukemia from hydrocele and to measure the testicular volume. Testicular size should also be measured and followed with the use of an orchiometer. Overt testicular leukemia at diagnosis per se is not an indication for testicular irradiation, as many patients can be successfully treated with chemotherapy, including high-dose methotrexate. Ultrasonogram should again be performed upon completion of remission induction. If testicular size is still abnormally enlarged, the sonogram should be repeated after consolidation treatment with high-dose methotrexate and mercaptopurine. Persistently enlarged testes after consolidation treatment will be biopsied. Testicular irradiation (24 Gy) will be administered in the rare patients with positive biopsies, after consultation with a radiotherapist.

Vincristine Neurotoxicity
The maximum single dose of vincristine must not exceed 2 mg. Mild vincristine toxicities (jaw pain, constipation, decreased deep tendon reflexes) are anticipated.

Loss of voice due to vocal cord paralysis may be a complication of vincristine toxicity but it must be differentiated from pharyngitis or Candida infection of the cord. If persistent, severe abdominal cramps, gait impairment or SIADH develop, the dose may be reduced to 1 mg/m^2. Only motor paralysis or typhlitis warrants discontinuation of vincristine.

Typhlitis

The best way to make a diagnosis is to perform ultrasonogram or CT. It occurs especially during consolidation, and can be fatal.

Venous thromboembolism

For patients who develop cerebral or other venous thrombosis will receive low molecular weight heparin throughout treatment with Asparaginase (during Reinduction in standard-risk patients, during the first 30 weeks of continuation treatment in high-/very high-risk cases, and during reintensification I in very high-risk patients).

In SR cases, during each reinduction treatment, dexamethasone will be given only in the first week and L-asparaginase in the second and third weeks (i.e., omit dexamethasone in the third week and L-asparaginase in the first week) and low molecular weight heparin will be given throughout reinduction treatment. In HR cases, dexamethasone will be omitted from weeks 4 and 9 and low molecular weight heparin will be given during the first 19 weeks of continuation treatment.

Avascular Necrosis of Bone

MRI exams will be interpreted by the radiologist. If there is evidence of epiphyseal or metaphyseal hip lesions, knee epiphyseal or metaphyseal lesions, or lesions of talus consistent with avascular necrosis of the bone, the patient will be referred to the orthopedic surgeon, who will evaluate symptoms, and will assess the severity and estimated risk of progression. Physical therapy, activity modifications, and surgical procedures will be recommended as needed. Patients with hip epiphyseal lesions or talus lesions affecting > 30% of weight-bearing area will have an X-ray of the affected area, and will be assessed at higher risk for progression. Symptomatic patients with such findings will likely have their dexamethasone stopped, especially if they are past reinduction II in therapy. Asymptomatic patients with such findings will likely have their dexamethasone dose halved, especially if they are past reinduction II in therapy. Any patients with X-ray findings of AVN are candidates for dexamethasone modification, regardless of symptoms. All modifications (or lack thereof) of dosage will be recorded in the research database. Patients with progression of any lesions or with

worsening symptoms will be re-evaluated by imaging and, if appropriate, by additional orthopedic follow-up. If the dexamethasone is discontinued, the, first choice will be to replace each week's dosing with one dose of methotrexate (40 mg/ m^2).

L-asparaginase Hypersensitivity

Patients with allergic reactions (e.g., severe pain and tenderness at injection site, urticaria) to *E. coli* L-asparaginase will be subsequently given *Erwinia* L-asparaginase. Erwinia L-asparaginase will be given at 20,000 U/m^2/dose during remission induction and, in SR cases, during reinduction; it will be given at 25,000 U/m^2 twice weekly (3 to 4 days apart) from weeks 1 to 19 of continuation treatment in HR cases. Patients allergic to both *E. coli* and *Erwinia* preparations will receive PEG-asparaginase (at 2500 U/m^2 per week). Acute hemorrhagic pancreatitis or severe pancreatitis (abdominal pain $\geq$72 hours and increased amylase $\geq$3 x normal) should be treated with Sandostatin, and is a contraindication to further L-asparaginase treatment. In the case of mild to moderate pancreatitis, asparaginase should be held until symptoms and signs subside, and amylase levels return to normal and then resumed. Asparaginase treatment should be delayed until at least 2 hours after intrathecal treatment. If patients become allergic to all three forms of asparaginase, the week of continuation treatment with asparaginase should constitute at least 6-mercaptopurine 50 mg/m^2/day and methotrexate 40 mg/m^2/week.

Pancreatitis

Acute hemorrhagic pancreatitis is a contraindication to continue asparaginase treatment. In the case of mild to moderate pancreatitis, asparaginase should be held until symptoms and signs subside, and amylase and lipase levels return to normal and then resumed. Any patients with abdominal pain suspected of pancreatitis should have serum amylase and lipase measured as well as an abdominal sonogram or CT scan done. In the case of severe pancreatitis (i.e. abdominal pain of 72 hours or more, amylase level three times or more of the upper limit of normal, and sonographic or CT scan evidence of pancreatitis), asparaginase may be discontinued permanently when the possibility of glucocorticoid- or mercaptopurine-induced pancreatitis is excluded. In cases with mild to moderate pancreatitis (abdominal pain less than 72 hours and amylase and lipase level less than three times the upper limit of normal), asparaginase should be held and resumed once symptoms and signs subsided. Call the PI or co-PI to discuss the management if the patient is asymptomatic (without abdominal pain) and has only elevated amylase or lipase levels. Consideration should also be given to dexamethasone- or mercaptopurine- related pancreatitis. Contact the PI or co-PI to discuss the management if there is a possiblility that the pancreatitis is due to either of

these two drugs.

Philadelphia Chromosome Positive (Ph+) ALL

Patients with Ph+ ALL will receive dasatinib (60 mg/ m^2 once daily.), starting on day 15 of induction chemotherapy. The dasatinib dose may be rounded up to the nearest 5 mg dose. If pleural effusion occurs, the dasatinib dose will be decreased by 25% but resumed at 60 mg/m^2 after recovery. If myelosuppression results in therapy interruption, the dosages of other myelosuppressive drugs should be reduced first.

7. DOSE MODIFICATIONS DURING CONTINUATION THERAPY

Dosage of continuation treatment should be titrated to keep WBC between 1800 and 3000/mm^3, ANC between 500 and 1200/mm^3 (with the exception of the count one week after dexamethasone treatment), and platelet count ≥ 50,000/mm^3.

Full dose of treatment will be administered when WBC ≥ 1500/ mm^3, ANC is ≥ 300/ mm^3 and platelet count ≥ 50,000/mm^3; except when dose reduction is clinically indicated. If patient is clinically well and ANC < 300/ mm^3, full dose of treatment can be administered if WBC ≥ 1500/ mm^3, platelet count ≥ 50,000/mm^3, and APC ≥ 500/ mm^3. Epirubicin, cyclophosphamide, cytarabine, methotrexate and mercaptopurine may be reduced by 30 to 50% if WBC is between 1000 and 1500/ mm^3 with ANC ≥ 300/ mm^3 and platelet count ≥ 50,000/mm^3. Disproportionate dose reduction of one agent compared to another should be avoided unless clinically indicated. Frequent (e.g., weekly) changes of mercaptopurine doses should be avoided.

Mercaptopurine and methotrexate will be reduced if WBC and ANC do not double a week after the start date of dexamethasone pulse. Exception can be made in patients whose WBC and ANC do not double but are over 3000 and 1200 respectively with no history of prior chemotherapy interruption or myelosuppression. In high-risk patients consider reducing cyclophosphamide and cytarabine dose if suspected to contribute to myelosuppression.

Dexamethasone, vincristine, and asparaginase will be given regardless of blood counts, provided that the patient is clinically well. On the weeks of vincristine and dexamethasone pulses, mercaptopurine may be reduced by 30% to 50% in patients with WBC < 1500/ mm^3 the week after prior dexamethasone pulse. In Standard Risk patients consider reducing cyclophosphamide and cytarabine dose if suspected to contribute to myelosuppression. Adjustments of dosages should be made in the following circumstances, with re-evaluation of tolerance and toxicities every 8 to 16 weeks

Dose Modifications for Inadequate Myelosuppression

Patients who miss less than 25% of therapy but have persistently (>50% of time; not counting the week after dexamethasone/vincristine) high WBC (>3,000/mm^3) and high ANC (>1000/ mm^3) should be counseled on compliance. If the WBC remains high, mercaptopurine and methotrexate dosages should be increased by 30% (e.g., to 100 mg/ m^2 and 50 mg/ m^2, respectively), using a stepwise approach if needed.

Dasatinib dosage adjustments (Ph+ ALL patients only)

Consider holding dasatinib for grade III-IV non-hematological toxicity until resolution to grade I or less. Hold dasatinib for pleural effusion. Consider replacing with imatinib 340 mg/ m^2 daily (as given on TXV) if patient cannot tolerate dasatinib. All modifications should be discussed with PI or co-PI.

Avoid concurrent use of drugs that are generally accepted to have a risk of causing QT interval prolongation or so called Torsades de Pointes (including quinidine, clarithromycin, erythromycin, chlorpromazine, haloperidol, arsenic, chloroquine, domperidone, pentamidine, pseudoephedrine), and drugs known to be interfere with CYP3A4.

Discontinue Bactrim when ANC < 200/mm^3.

8. PATIENT EVALUATION

Pretreatment Evaluation

1. Complete history and physical exam with careful notation and assessment of clinical signs relevant to leukemia: liver, spleen, lymph nodes, gum or skin infiltration, local or systemic infections, testes
2. Complete blood count, differential and platelet count
3. Chemical profile: glucose, BUN, creatinine, LDH, uric acid, Alk-P, bilirubin, SGOT, SGPT, calcium, phosphorous, sodium, potassium, total protein, and albumin
4. Hepatitis B surface antigen
5. Lumbar puncture with CSF routine examination and cytospin
6. Bone marrow evaluation for cytomorphology and cytochemistries, surface markers, cytogenetics, DNA index and molecular assay (if available)
7. Chest X-Ray (PA and/or lateral)
8. Blood culture for all febrile patients
9. Other clinical investigations including echocardiogram, computed tomography, sinus films, etc., if clinically indicated

Post-treatment Evaluation

1. Postprandial blood sugar, albumin, amylase, lipase BiW during L-asparaginase therapy
2. Sodium QW in induction therapy (watching for SIADH)
3. Chemical profile, if needed, esp. within 48 hours after chemotherapy is started.
4. BUN and creatine before and after HDMTX
5. CBC
6. Plasma MTX level after HDMTX therapy

Evaluation Criteria

1. Complete remission: M1 marrow status with restoration of normal hematopoiesis and normal performance status. These findings must persist for a least one month.
2. Induction failure: ≥ 5% leukemic blasts in marrow after 42 days of remission induction treatment.
3. Bone marrow relapse: ≥ 25% leukemic blasts in marrow
4. CNS relapse: ≥ 5 WBC/μL of CSF with definite blasts on cytospin preparation
5. Testicular relapse: Isolated testicular relapse must be confirmed pathologically; in the event of bone marrow relapse, combined testicular relapse can be based on testicular enlargement (documented by sonogram) without biopsy.

Patient Exclusions

Except for the patients who are entered but subsequently found not to have met the eligibility criteria, all patients will be included in all analyses. Thus, if a patient achieves remission but is subsequently removed from the study because of toxicity, the outcome (time to failure, survival, ect.) for that patient is included in analysis. Patients removal from study because of refusal of therapy could be censored at the time.

Primary End-Points (definitions)

There are eight primary end-points that are commonly used in the analysis of leukemia studies. These are listed and defined below. Seven of the eight end-points measure the time from some starting point to the time of "failure" so that methods for handling censored data must be used. (A censored observation occurs when a patient has not yet failed.)

Complete Remission (CR) – This is a "yes-no" variable that refers to a complete remission attained on the specified induction regimen. If a patient is taken off study prior to attaining a CR, then that patient is counted as a non-responder, even if a CR was achieved later on a different therapy.

Time to Failure (event-free survival) – Time from date of attaining CR to first failure of any kind for patients with CR to induction therapy; zero time length for nonresponders. All failures are counted, including death in CR or second malignancy, except basal cell carcinoma and in situ carcinoma.

Length of Survival – Time from on-study date to death date; time from on-study date to last follow-up for those who are still alive.

Length of Complete Remisison – Time from initial CR date to initial failure or relapse date; time from initial CR date to last known date in CR for patients who have not failed or relapsed. Deaths in CR and second malignancies are counted as remission failures with respect to length of CR (as in definition of time to failure).

Length of Hematologic Remission (HR) – Time from initial CR date to the date of first hematologic relapse, or to last follow-up date for patients who have not had a hematologic relapse. A death or second malignancy prior to a hematologic relapse is counted as a censored observation with respect to length of HR. Nonhematologic relapses (CNS, testicular or other relapses) are not counted as failures and are ignored for the analysis of this end-point.

Length of CNS Remission – Time from the initial CR date to the date of CNS relapse, or to last follow-up date for patients who have not had a CNS relapse. A death or second malignancy prior to a CNS relapse is counted as a censored observation with respect to length of CNS remission. Non-CNS relapses (hematologic, testicular or other relapses)

are not counted as failures and are ignored for the analysis of length of CNS remission.

Time to Isolated CNS Relapse – This is the same as the length of CNS remission if no hematologic relapse has occurred prior to or concurrently with the CNS relapse. If a hematologic relapse has occurred, this is the same as the hematologic remission duration and the event is censored at the time of hematologic relapsed. Other relapses (e.g., testicular) are also counted as censored obsevations.

Time to Testicular Relapse – Time from the initial CR date to the date of testicular relapse, or the last follow-up date for boys who have not had a testicular relapse. A death or second malignancy prior to a testicular relapse is counted as a censored observation with respect to time to testicular relapse. Non-testicular relapses (hematologic or other) are not counted as failures and are ignored for the analysis of time to testicular relapse.

9. CONTINGENCY PLANS FOR REFRACTORY DISEASE OR RELAPSE

Induction failures

Patients who do not attain complete remission (≥ 5% leukemic blasts in bone marrow) after remission induction, consolidation treatment and reintensification treatment will be removed from the protocol. Those who do not achieve a remission after induction therapy, but subsequently attain complete remission after consolidation or reintensification treatment, are candidates for allogeneic hematopoietic stem cell transplantation.

Hematologic relapse

Patients with ≥ 25% lymphoblasts in marrow aspirate will become eligible for relapse protocols.

Extramedullary relapse

Patients with any form of extramedullary relapse (testes, ovarian, etc) except that of CNS will become eligible for relapse protocols. Patients with overt CNS relapse (i.e. ≥ 5 WBC/μL of CSF with blasts) will remain on study and receive treatment outlined in Section 9.4. Patients who have <5 WBC/μL of CSF with identifiable blasts are not considered to have overt CNS relapse and will be treated as outlined in Section 9.4.

Emergence of CSF Lymphoblasts During Remission Requiring CNS Radiation:

Preventive cranial irradiation will not be given prophylactically to patients with CNS leukemia at diagnosis or to those with high-risk leukemia. Only patients with immunologically proven leukemic lymphoblasts in CSF (regardless of cell count) during hematologic remission will receive therapeutic CNS irradiation after receiving a second remission induction followed by 1-2 cycles of reintensification (reinduction) to consolidate bone marrow remission after induction. Whether 1 or 2 cycles are to be given will depend on risk group, time to emergence of blasts in CSF, immunophenotype of blasts, and individual patient tolerance. In general, high/very-high risk patients or those with early occurrence (<18 months) will receive 2 cycles. Triple intrathecal therapy will be continued every 3 to 4 weeks before CNS irradiation

CNS irradiation for patients with CNS relapse is indicated as follows:

1.Cranial irradiation (18 Gy in 12 fractions) for those with any number of leukemic lymphoblasts in CSF after 18 months of initial remission

2.Cranial irradiation (24 Gy in 16 fractions) for patients with <5 WBC/μL of CSF

occurring within the first 18 months of remission

3.Craniospinal irradiation (24 Gy cranial irradiation in 16 fractions plus 15 Gy spinal irradiation in 10 fractions) for patients with ≥ 5 WBC/μL of CSF occurring within the first 18 months of remission.

Those patients receiving cranial irradiation only should receive 4 to 5 triple intrathecal therapy with leucovorin rescue during irradiation. No TIT will be given after cranial irradiation.

Mercaptopurine and methotrexate will be withheld for at least one week prior to and during irradiation.

Systemic chemotherapy during irradiation will include dexamethasone and vincristine with or without L-asparaginase.

Continuation treatment will be given for at least one year from time of relapse (or at least 2½ years from the start of treatment).

10. SUPPORTIVE CARE

Fever at Diagnosis

All patients with fever at diagnosis will be admitted for broad spectrum parenteral antibiotic treatment until an infectious etiology can be excluded.

Metabolic Derangements

It is important to prevent or treat hyperuricemia and hyperphosphatemia with secondary hypocalcemia resulting from spontaneous or chemotherapy-induced leukemic cell lysis, especially in T-cell ALL.

Patients with large leukemic cell burden should receive hydration and oral phosphate binder.

Patients with large leukemic cell burden with or without hyperuricemia (e.g., WBC ≥ 100,000/mm^3, uric acid ≥ 7.5 mg/dl or ≥ 6.5 mg/dl in patients <13 years old) may be treated with rasburicase if they have no history of G6PD deficiency or ongoing pregnancy. Patients with history of severe allergy (e.g., bronchial asthma requiring bronchodilator, atopic eczema), may be enrolled on RASALL. For all other patients not at high risk of hyperuricemia, hydration, allopurinol, and judicious use of alkalinization (keeping urine pH between 6.5 and 7.4) may be sufficient.

Hyperleukocytosis

For patients with extreme hyperleukocytosis (i.e., WBC ≥ 300,000/mm^3), leukapheresis or exchange transfusion (in small children) may be considered. The Director of the Blood Bank should be consulted for this procedure.

Avascular Necrosis of Bone

Osteonecrosis of the bone, a known complication of treatment with corticosteroids, can be expected to occur in approximately 10-15% of patients, especially in those older than nine years of age. This devastating complication may result in collapse of the articulating surface with subsequent pain and development of arthritis. Early detection of small lesions will permit intervention which may prevent pain and irreversible damage of the joints. In this study, all patients 9 years of age and older will have MRI scans of the pelvis/hips and knees after each reinduction phase, at off therapy date, and as needed thereafter. Patients diagnosed with osteonecrosis will be referred to orthopedics. Any patient who develops symptoms of joint pain prior to or between scheduled MRI scans should have an MRI performed to rule out osteonecrosis or progression of this complication.

For patients who require surgical intervention, treatment will vary based on degree of progression, i.e., observation, core decompression, bone grafting and resurfacing hemiarthroplasty.

Pancytopenias

Patients with prolonged (> 3 weeks) unexplained anemia (hemoglobin < 7 g/dl) or neutropenia (ANC < 300/mm^3) during remission should be evaluated for B19 parvovirus infection or hemolysis or toxicity from non-chemotherapeutic agents (e.g., TMP/SMZ).

Nutritional Supplementation

Nutritional or vitamin therapies should not result in patients receiving more than the RDA for folic acid with dietary and supplement intake, to prevent interference with the effectiveness of methotrexate.

Drug Interactions

Because concurrent use of enzyme inducing anticonvulsants (e.g. phenytoin, phenobarbital, and carbamazepine) with antileukemic therapy has recently been associated with inferior EFS, every effort should be made to avoid these agents, as well as rifampin, which also induces many drug metabolizing enzymes. Gabapentin does not induce hepatic drug metabolizing enzymes and may be a suitable alternative anticonvulsant.

Azole antifungals (fluconazole, itraconazole, and ketoconazole) and the macrolide antibiotics (erythromycin, rifampin, and zithromax) may have potent inhibitory effects on drug-metabolizing enzymes, and the doses of some antileukemic drugs (e.g. vincristine, anthracyclines, etoposide) may need to be reduced in some patients on chronic treatments.

Penicillins interfere with tubular excretion of methotrexate, and it is recommended that an alternative non-penicillin antibiotic be used.

Down Syndrome

Patients with Down's syndrome should be closely monitored for toxicity, and offered aggressive supportive care. Methotrexate dosage will be reduced as described in section 6. Historically, most of these patients will be treated in the low-risk category, and should have their chemotherapy doses appropriately reduced to avoid undue toxicity. Oral leucovorin (5 mg/ m^2 q 12 hr x 2) should be given 24 hrs after each IT MHA. A 30% dose reduction of dexamethasone and/or high dose cytarabine should be considered for

Down's syndrome patients treated on the standard/high-risk arm who experience higher than expected toxicity during earlier phases of therapy.

RSV prophylaxis
All infants should receive RSV prophylaxis as per current institutional policy.

Prophylactic antibiotics during periods of prolonged neutropenia
Patients with expected periods of severe neutropenia (ANC<500) of 7 days or longer (e.g. during induction, reinduction, and intensification) should receive prophylactic antibiotics (ciprofloxacin 250-350 mg/m^2/12h plus vancomycin 400 mg/m^2/12h) and antifungals (micafungin, 2mg/kg/day with max. 50mg/day, if patient is receiving weekly vincristine, or voriconazole 4mg/kg/12h if patient has completed all vincristine doses in the treatment phase).

11. TOXICITY AND COMPLICATIONS CRITERIA

Toxicity of chemotherapy will be evaluated using the ECOG guidelines (see Appendix I of TPOG ALL-93 protocol)

Definitions of infection (see Appendix II of TPOG ALL-93 protocol)

12.DROP OFF CRITERIA

1.Incorrect diagnosis.
2.Patient and/or parents refuse to allow additional therapy.
3.A patient who, in the judgement of the Principal Investigator, could not or did not follow the assigned treatment, may be removed from study.
4.Patients who fail to meet all eligibility requirements of protocol (i.e., ineligible) will be taken off study, e.g., using other protocols, or not newly diagnosed patients.

13. REFERENCES

1. Nachman JB, Sather HN, Sensel MG et al. Augmented post-induction therapy for children with high-risk acute lymphoblastic leukemia and a slow response to initial therapy. N.Engl.J.Med. 1998;338:1663-1671.
2. Silverman LB, Gelber RD, Dalton VK et al. Improved outcome for children with acute lymphoblastic leukemia: results of Dana-Farber Consortium Protocol 91-01. Blood 2001;97:1211-1218.
3. Vora A, Mitchell CD, Lennard L et al. Toxicity and efficacy of 6-thioguanine versus 6-mercaptopurine in childhood lymphoblastic leukaemia: a randomised trial. Lancet 2006;368:1339-1348.
4. Mitchell CD, Richards SM, Kinsey SE et al. Benefit of dexamethasone compared with prednisolone for childhood acute lymphoblastic leukaemia: results of the UK Medical Research Council ALL97 randomized trial. Br.J.Haematol. 2005;129:734-745.
5. Bostrom BC, Sensel MR, Sather HN et al. Dexamethasone versus prednisone and daily oral versus weekly intravenous mercaptopurine for patients with standard-risk acute lymphoblastic leukemia: a report from the Children's Cancer Group. Blood 2003;101:3809-3817.
6. Matloub Y, Lindemulder S, Gaynon PS et al. Intrathecal triple therapy decreases central nervous system relapse but fails to improve event-free survival when compared with intrathecal methotrexate: results of the Children's Cancer Group (CCG) 1952 study for standard-risk acute lymphoblastic leukemia, reported by the Children's Oncology Group. Blood 2006;108:1165-1173.
7. Pinheiro JP, Boos J. The best way to use asparaginase in childhood acute lymphatic leukaemia--still to be defined? Br.J.Haematol. 2004;125:117-127.
8. Wetzler M, Sanford BL, Kurtzberg J et al. Effective asparagine depletion with pegylated asparaginase results in improved outcomes in adult acute lymphoblastic leukemia: Cancer and Leukemia Group B Study 9511. Blood 2007;109:4164-4167.
9. Hijiya N, Franklin J, Rytting M et al. A phase I study of clofarabine in combination with cyclophosphamide and etoposide: A new regimen in pediatric patients with refractory or relapsed acute leukemia. ASCO Meeting Abstracts 2007;25:9529.
10. Flotho C, Coustan-Smith E, Pei D et al. Genes contributing to minimal residual disease in childhood acute lymphoblastic leukemia: prognostic significance of CASP8AP2. Blood 2006;108:1050-1057.
11. Flotho C, Coustan-Smith E, Pei D et al. A set of genes that regulate cell proliferation predicts treatment outcome in childhood acute lymphoblastic leukemia. Blood 2007;110:1271-1277.
12. Flotho C, Coustan-Smith E, Pei D et al. Genes contributing to minimal residual

disease in childhood acute lymphoblastic leukemia: prognostic significance of CASP8AP2. Blood 2006;108:1050-1057.
13. Holleman A, Cheok MH, den Boer ML et al. Gene-expression patterns in drug-resistant acute lymphoblastic leukemia cells and response to treatment. N.Engl.J.Med. 2004;351:533-542.
14. Lugthart S, Cheok MH, den Boer ML et al. Identification of genes associated with chemotherapy crossresistance and treatment response in childhood acute lymphoblastic leukemia. Cancer Cell 2005;7:375-386.
15. Flotho C, Coustan-Smith E, Pei D et al. A set of genes that regulate cell proliferation predicts treatment outcome in childhood acute lymphoblastic leukemia. Blood 2007;110:1271-1277.
16. Flotho C, Coustan-Smith E, Pei D et al. A set of genes that regulate cell proliferation predicts treatment outcome in childhood acute lymphoblastic leukemia. Blood 2007;110:1271-1277.
17. Pui CH, Evans WE. Treatment of acute lymphoblastic leukemia. N.Engl.J.Med. 2006;354:166-178.
18. Pui CH, Relling MV, Downing JR. Acute lymphoblastic leukemia. N.Engl.J.Med. 2004;350:1535-1548.
19. Hilden JM, Dinndorf PA, Meerbaum SO et al. Analysis of prognostic factors of acute lymphoblastic leukemia in infants: report on CCG 1953 from the Children's Oncology Group. Blood 2006;108:441-451.
20. Pieters R, Schrappe M, De LP et al. A treatment protocol for infants younger than 1 year with acute lymphoblastic leukaemia (Interfant-99): an observational study and a multicentre randomised trial. Lancet 2007;370:240-250.
21. Pui CH, Campana D, Evans WE. Childhood acute lymphoblastic leukaemia--current status and future perspectives. Lancet Oncol. 2001;2:597-607.
22. Pui CH, Evans WE. Acute lymphoblastic leukemia. N.Engl.J.Med. 1998;339:605-615.
23. Coustan-Smith E, Sancho J, Behm FG et al. Prognostic importance of measuring early clearance of leukemic cells by flow cytometry in childhood acute lymphoblastic leukemia. Blood 2002;100:52-58.
24. Pui CH. Central nervous system disease in acute lymphoblastic leukemia: prophylaxis and treatment. Hematology.Am.Soc.Hematol.Educ.Program. 2006142-146.
25. Pui CH, Sandlund JT, Pei D et al. Improved outcome for children with acute lymphoblastic leukemia: results of Total Therapy Study XIIIB at St Jude Children's Research Hospital. Blood 2004;104:2690-2696.
26. Pui CH, Gaynon PS, Boyett JM et al. Outcome of treatment in childhood acute lymphoblastic leukaemia with rearrangements of the 11q23 chromosomal region.

Lancet 2002;359:1909-1915.
27. Arico M, Valsecchi MG, Camitta B et al. Outcome of treatment in children with Philadelphia chromosome-positive acute lymphoblastic leukemia. N.Engl.J.Med. 2000;342:998-1006.
28. Talpaz M, Shah NP, Kantarjian H et al. Dasatinib in imatinib-resistant Philadelphia chromosomepositive leukemias. N.Engl.J.Med. 2006;354:2531-2541.
29. Ottmann O, Dombret H, Martinelli G et al. Dasatinib induces rapid hematologic and cytogenetic responses in adult patients with Philadelphia chromosome-positive acute lymphoblastic leukemia with resistance or intolerance to imatinib: interim results of a Phase II study. Blood 2007blood- 2007.
30. Kantarjian H, Pasquini R, Hamerschlak N et al. Dasatinib or high-dose imatinib for chronic-phase chronic myeloid leukemia after failure of first-line imatinib: a randomized phase 2 trial. Blood 2007;109:5143-5150.
31. Coustan-Smith E, Mullighan CG, Onciu M et al. Early T-cell precursor leukaemia: a subtype of very high-risk acute lymphoblastic leukaemia. Lancet Oncol 2009;10:147-156.
32. Schrappe M, Zimmermann M, Moricke A et al. Dexamethasone in Induction Can Eliminate One Third of All Relapses in Childhood Acute Lymphoblastic Leukemia (ALL): Results of An International Randomized Trial in 3655 Patients (Trial AIEOP-BFM ALL 2000). ASH Annual Meeting Abstracts 2008;112:7.
33. Reiter A, Schrappe M, Ludwig WD et al. Chemotherapy in 998 unselected childhood acute lymphoblastic leukemia patients. Results and conclusions of the multicenter trial ALL-BFM 86. Blood 1994;84:3122-3133.
34. Conter V, Arico M, Valsecchi MG et al. Intensive BFM chemotherapy for childhood ALL: interim analysis of the AIEOP-ALL 91 study. Associazione Italiana Ematologia Oncologia Pediatrica. Haematologica 1998;83:791-799.
35. Galpin AJ, Schuetz JD, Masson E et al. Differences in folylpolyglutamate synthetase and dihydrofolate reductase expression in human B-lineage versus T-lineage leukemic lymphoblasts: mechanisms for lineage differences in methotrexate polyglutamylation and cytotoxicity. Mol.Pharmacol. 1997;52:155-163.
36. Synold TW, Relling MV, Boyett JM et al. Blast cell methotrexate-polyglutamate accumulation in vivo differs by lineage, ploidy, and methotrexate dose in acute lymphoblastic leukemia. J.Clin.Invest 1994;94:1996-2001.
37. Niemeyer CM, Gelber RD, Tarbell NJ et al. Low-dose versus high-dose methotrexate during remission induction in childhood acute lymphoblastic leukemia (Protocol 81-01 update). Blood 1991;78:2514-2519.
38. Evans WE, Schell MJ, Pui CH. MTX clearance is more important for intermediate-risk ALL. J.Clin.Oncol. 1990;8:1115-1116.

39. Wall AM, Gajjar A, Link A et al. Individualized methotrexate dosing in children with relapsed acute lymphoblastic leukemia. Leukemia 2000;14:221-225.
40. Evans WE, Relling MV, Rodman JH et al. Conventional compared with individualized chemotherapy for childhood acute lymphoblastic leukemia. N.Engl.J.Med. 1998;338:499-505.
41. Bostrom B, Gaynon PS, Sather H et al. Dexamethasone (DEX) decreases central nervous system (CNS) relapse and improves event-free survival (EFS) in lower risk acute lymphoblastic leukemia (ALL) (Meeting abstract). [abstract]. Am.Soc.of Clinical Oncology 1998;17:5279.
42. Relling MV, Hancock ML, Boyett JM, Pui CH, Evans WE. Prognostic importance of 6- mercaptopurine dose intensity in acute lymphoblastic leukemia. Blood 1999;93:2817-2823.
43. Silverman LB, Gelber RD, Dalton VK et al. Improved outcome for children with acute lymphoblastic leukemia: results of Dana-Farber Consortium Protocol 91-01. Blood 2001;97:1211-1218.
44. Nachman JB, Sather HN, Sensel MG et al. Augmented post-induction therapy for children with high-risk acute lymphoblastic leukemia and a slow response to initial therapy. N.Engl.J.Med. 1998;338:1663-1671.
45. Amylon MD, Shuster J, Pullen J et al. Intensive high-dose asparaginase consolidation improves survival for pediatric patients with T cell acute lymphoblastic leukemia and advanced stage lymphoblastic lymphoma: a Pediatric Oncology Group study. Leukemia 1999;13:335-342.
46. Pui CH, Relling MV, Behm FG et al. L-asparaginase may potentiate the leukemogenic effect of the epipodophyllotoxins. Leukemia 1995;9:1680-1684.
47. Pui CH, Relling MV, Behm FG et al. L-asparaginase may potentiate the leukemogenic effect of the epipodophyllotoxins. Leukemia 1995;9:1680-1684.
48. Nysom K, Holm K, Lipsitz SR et al. Relationship between cumulative anthracycline dose and late cardiotoxicity in childhood acute lymphoblastic leukemia. J.Clin.Oncol. 1998;16:545-550.
49. Nachman JB, Sather HN, Sensel MG et al. Augmented post-induction therapy for children with high-risk acute lymphoblastic leukemia and a slow response to initial therapy. N.Engl.J.Med. 1998;338:1663-1671.
50. Evans WE, Relling MV, Rodman JH et al. Conventional compared with individualized chemotherapy for childhood acute lymphoblastic leukemia. N.Engl.J.Med. 1998;338:499-505.
51. Ludwig WD, Rieder H, Bartram CR et al. Immunophenotypic and genotypic features, clinical characteristics, and treatment outcome of adult pro-B acute lymphoblastic leukemia: results of the German multicenter trials GMALL 03/87 and

04/89. Blood 1998;92:1898-1909.
52. Jeha S, Pei D, Raimondi SC et al. Increased risk for CNS relapse in pre-B cell leukemia with the t(1;19)/TCF3-PBX1. Leukemia 2009;23:1406-1409.
53. Yeoh EJ, Ross ME, Shurtleff SA et al. Classification, subtype discovery, and prediction of outcome in pediatric acute lymphoblastic leukemia by gene expression profiling. Cancer Cell 2002;1:133-143.
54. Rocha JC, Cheng C, Liu W et al. Pharmacogenetics of outcome in children with acute lymphoblastic leukemia. Blood 2005;105:4752-4758.
55. Cheok MH, Evans WE. Acute lymphoblastic leukaemia: a model for the pharmacogenomics of cancer therapy. Nat.Rev.Cancer 2006;6:117-129.
56. Evans WE, Relling MV. Moving towards individualized medicine with pharmacogenomics 1. Nature 2004;429:464-468.
57. Relling MV, Pui CH, Cheng C, Evans WE. Thiopurine methyltransferase in acute lymphoblastic leukemia. Blood 2006;107:843-844.
58. Holleman A, Cheok MH, den Boer ML et al. Gene-expression patterns in drug-resistant acute lymphoblastic leukemia cells and response to treatment. N.Engl.J.Med. 2004;351:533-542.
59. Bogni A, Cheng C, Liu W et al. Genome-wide approach to identify risk factors for therapyrelated myeloid leukemia. Leukemia 2006;20:239-246.
60. Cheok MH, Yang W, Pui CH et al. Treatment-specific changes in gene expression discriminate in vivo drug response in human leukemia cells. Nat.Genet. 2003;34:85-90.
61. Yang JJ, Cheng C, Yang W et al. Genome-wide Interrogation of Germline Genetic Variation Associated With Treatment Response in Childhood Acute Lymphoblastic Leukemia. JAMA: The Journal of the American Medical Association 2009;301:393-403.
62. Relling MV, Yang W, Das S et al. Pharmacogenetic Risk Factors for Osteonecrosis of the Hip Among Children With Leukemia. J Clin Oncol 2004;22:3930-3936.
63. Yang J, Cheng C, Yang W et al. Genome-wide germline variation and treatment response in acule lymphoblastic leukemia (ALL) [abstract]. Am J Hum Genet 2007;Meeting Abstract:
64. Kishi S, Cheng C, French D et al. Ancestry and pharmacogenetics of antileukemic drug toxicity. Blood 2007;109:4151-4157.
65. Kishi S, Yang W, Boureau B et al. Effects of prednisone and genetic polymorphisms on etoposide disposition in children with acute lymphoblastic leukemia. Blood 2004;103:67-72.
66. Kishi S, Griener J, Cheng C et al. Homocysteine, Pharmacogenetics, and Neurotoxicity in Children With Leukemia. J Clin Oncol 2003;21:3084-3091.

67. Blanco JG, Edick MJ, Hancock ML et al. Genetic polymorphisms in CYP3A5, CYP3A4 and NQO1 in children who developed therapy-related myeloid malignancies. Pharmacogenetics 2002;12:605-611.
68. Relling MV, Yang W, Das S et al. Pharmacogenetic Risk Factors for Osteonecrosis of the Hip Among Children With Leukemia. J Clin Oncol 2004;22:3930-3936.
69. Cheok MH, Evans WE. Acute lymphoblastic leukaemia: a model for the pharmacogenomics of cancer therapy. Nat.Rev.Cancer 2006;6:117-129.
70. Holleman A, Cheok MH, den Boer ML et al. Gene-expression patterns in drug-resistant acute lymphoblastic leukemia cells and response to treatment. N.Engl.J.Med. 2004;351:533-542.
71. Bogni A, Cheng C, Liu W et al. Genome-wide approach to identify risk factors for therapyrelated myeloid leukemia. Leukemia 2006;20:239-246.
72. Cheok MH, Yang W, Pui CH et al. Treatment-specific changes in gene expression discriminate in vivo drug response in human leukemia cells. Nat.Genet. 2003;34:85-90.
73. Mullighan CG, Goorha S, Radtke I et al. Genome-wide analysis of genetic alterations in acute lymphoblastic leukaemia. Nature 2007;446:758-764.
74. Cheng Q, Yang W, Raimondi SC et al. Karyotypic abnormalities create discordance of germline genotype and cancer cell phenotypes. Nat.Genet. 2005;37:878-882.
75. Weinshilboum RM, Sladek SL. Mercaptopurine pharmacogenetics: monogenic inheritance of erythrocyte thiopurine methyltransferase activity. Am.J.Hum.Genet. 1980;32:651-662.
76. Avramis VI, Sencer S, Periclou AP et al. A randomized comparison of native Escherichia coli asparaginase and polyethylene glycol conjugated asparaginase for treatment of children with newly diagnosed standard-risk acute lymphoblastic leukemia: a Children's Cancer Group study. Blood 2002;99:1986-1994.
77. Vieira Pinheiro JP, Wenner K, Escherich G et al. Serum asparaginase activities and asparagine concentrations in the cerebrospinal fluid after a single infusion of 2,500 IU/m(2) PEG asparaginase in children with ALL treated according to protocol COALL-06-97. Pediatr.Blood Cancer 2006;46:18-25.
78. Yang L, Panetta JC, Cai X et al. Asparaginase may influence dexamethasone pharmacokinetics in acute lymphoblastic leukemia. J Clin Oncol 2008;26:1932-1939.
79. Holleman A, Cheok MH, den Boer ML et al. Gene-expression patterns in drug-resistant acute lymphoblastic leukemia cells and response to treatment. N.Engl.J.Med. 2004;351:533-542.
80. Lugthart S, Cheok MH, den Boer ML et al. Identification of genes associated with chemotherapy crossresistance and treatment response in childhood acute lymphoblastic leukemia. Cancer Cell 2005;7:375-386.

81. Holleman A, Cheok MH, den Boer ML et al. Gene-expression patterns in drug-resistant acute lymphoblastic leukemia cells and response to treatment. N.Engl.J.Med. 2004;351:533-542.
82. Lugthart S, Cheok MH, den Boer ML et al. Identification of genes associated with chemotherapy crossresistance and treatment response in childhood acute lymphoblastic leukemia. Cancer Cell 2005;7:375-386.
83. Holleman A, Cheok MH, den Boer ML et al. Gene-expression patterns in drug-resistant acute lymphoblastic leukemia cells and response to treatment. N.Engl.J.Med. 2004;351:533-542.
84. Lugthart S, Cheok MH, den Boer ML et al. Identification of genes associated with chemotherapy crossresistance and treatment response in childhood acute lymphoblastic leukemia. Cancer Cell 2005;7:375-386.
85. Wei G, Twomey D, Lamb J et al. Gene expression-based chemical genomics identifies rapamycin as a modulator of MCL1 and glucocorticoid resistance. Cancer Cell 2006;10:331-342.
86. Cheok MH, Yang W, Pui CH et al. Treatment-specific changes in gene expression discriminate in vivo drug response in human leukemia cells. Nat.Genet. 2003;34:85-90.
87. Holleman A, Cheok MH, den Boer ML et al. Gene-expression patterns in drug-resistant acute lymphoblastic leukemia cells and response to treatment. N.Engl.J.Med. 2004;351:533-542.
88. Lugthart S, Cheok MH, den Boer ML et al. Identification of genes associated with chemotherapy crossresistance and treatment response in childhood acute lymphoblastic leukemia. Cancer Cell 2005;7:375-386.
89. Campana D. Determination of minimal residual disease in leukaemia patients. Br.J.Haematol. 2003;121:823-838.
90. Coustan-Smith E, Sancho J, Behm FG et al. Prognostic importance of measuring early clearance of leukemic cells by flow cytometry in childhood acute lymphoblastic leukemia. Blood 2002;100:52-58.
91. Imai K, Matsuyama S, Miyake S, Suga K, Nakachi K. Natural cytotoxic activity of peripheralblood lymphocytes and cancer incidence: an 11-year follow-up study of a general population. Lancet 2000;356:1795-1799.
92. Costello RT, Sivori S, Marcenaro E et al. Defective expression and function of natural killer celltriggering receptors in patients with acute myeloid leukemia. Blood 2002;99:3661-3667.
93. Fauriat C, Just-Landi S, Mallet F et al. Deficient expression of NCR in NK cells from acute myeloid leukemia: Evolution during leukemia treatment and impact of leukemia cells in NCRdull phenotype induction. Blood 2007;109:323-330.

94. Pende D, Spaggiari GM, Marcenaro S et al. Analysis of the receptor-ligand interactions in the natural killer-mediated lysis of freshly isolated myeloid or lymphoblastic leukemias: evidence for the involvement of the Poliovirus receptor (CD155) and Nectin-2 (CD112). Blood 2005;105:2066-2073.
95. Leung W, Iyengar R, Turner V et al. Determinants of antileukemia effects of allogeneic NK cells. J.Immunol. 2004;172:644-650.
96. Fauriat C, Just-Landi S, Mallet F et al. Deficient expression of NCR in NK cells from acute myeloid leukemia: Evolution during leukemia treatment and impact of leukemia cells in NCRdull phenotype induction. Blood 2007;109:323-330.
97. Fauriat C, Just-Landi S, Mallet F et al. Deficient expression of NCR in NK cells from acute myeloid leukemia: Evolution during leukemia treatment and impact of leukemia cells in NCRdull phenotype induction. Blood 2007;109:323-330.
98. Flotho C, Coustan-Smith E, Pei D et al. Genes contributing to minimal residual disease in childhood acute lymphoblastic leukemia: prognostic significance of CASP8AP2. Blood 2006;108:1050-1057.
99. Flotho C, Coustan-Smith E, Pei D et al. A set of genes that regulate cell proliferation predicts treatment outcome in childhood acute lymphoblastic leukemia. Blood 2007;110:1271-1277.
100. Flotho C, Coustan-Smith E, Pei D et al. Genes contributing to minimal residual disease in childhood acute lymphoblastic leukemia: prognostic significance of CASP8AP2. Blood 2006;108:1050-1057.
101. Flotho C, Coustan-Smith E, Pei D et al. A set of genes that regulate cell proliferation predicts treatment outcome in childhood acute lymphoblastic leukemia. Blood 2007;110:1271-1277.
102. Flotho C, Coustan-Smith E, Pei D et al. Genes contributing to minimal residual disease in childhood acute lymphoblastic leukemia: prognostic significance of CASP8AP2. Blood 2006;108:1050-1057.
103. Holleman A, Cheok MH, den Boer ML et al. Gene-expression patterns in drug-resistant acute lymphoblastic leukemia cells and response to treatment. N.Engl.J.Med. 2004;351:533-542.
104. Lugthart S, Cheok MH, den Boer ML et al. Identification of genes associated with chemotherapy crossresistance and treatment response in childhood acute lymphoblastic leukemia. Cancer Cell 2005;7:375-386.
105. Flotho C, Coustan-Smith E, Pei D et al. A set of genes that regulate cell proliferation predicts treatment outcome in childhood acute lymphoblastic leukemia. Blood 2007;110:1271-1277.
106. Flotho C, Coustan-Smith E, Pei D et al. A set of genes that regulate cell proliferation predicts treatment outcome in childhood acute lymphoblastic leukemia. Blood

2007;110:1271-1277.
107. Reddick WE, Glass JO, Helton KJ et al. Leukoencephalopathy prevalence in children treated for acute lymphoblastic leukemia with high-dose methotrexate. Am J Neuroradiol 2005;2615:1263- 1269.
108. Reddick WE, Glass JO, Helton KJ et al. A quantitative MRI assessment of leukoencephalopathy in children treated for acute lymphoblastic leukemia without irradiation. Am J Neuroradiol 2005;26:2371-2377.
109. Toga AW, Thompson PM, Sowell ER. Mapping brain maturation. Trends Neurosci 2006;29:148-159.
110. Sowell ER, Thompson PM, Leonard CM et al. Longitudinal mapping of cortical thickness and brain growth in normal children. The Journal of Neuroscience 2004;24:8223-8231.
111. Khong P-L, Leung LHT, Fung ASM et al. White matter anisotropy in post-treatment childhood cancer survivors: preliminary evidence of association with neurocognitive function. J Clin Oncol 2006;24:884-890.
112. Montour-Proulx I, Kuehn SM, Keene DL et al. Cognitive changes in children treated for acute lymphoblastic leukemia with chemotherapy only according to the Pediatric Oncology Group 9605 protocol. J Child Neurol 2005;20:129-133.
113. Espy KA, Moore IM, Kaufmann PM et al. Chemotherapeutic CNS Prophylaxis and Neuropsychologic Change in Children With Acute Lymphoblastic Leukemia: A Prospective Study. J Pediatr Psychol 2001;26:1-9.
114. Kaemingk KL, Carey ME, Moore IM, Herzer M, Hutter JJ. Math weaknesses in survivors of acute lymphoblastic leukemia compared to healthy children. Child Neuropsychology 2004;10:14- 23.
115. Carey ME, Hockenberry M, Moore IM et al. Brief Report: Effect of intravenous methotrexate dose and infusion rate on neuropsychological function one year after diagnosis of acute lymphoblastic leukemia. J Pediatr Psychol 2007;32:189-193.
116. Brown RT, Madan-Swain A, Pais R et al. Chemotherapy for acute lymphocytic leukemia: cognitive and academic sequelae. J Pediatr 1992;121:885-889.
117. Paakko E, Harila-Saari A, Vanionpaa L et al. White matter changes on MRI during treatment in children with acute lymphoblastic leukemia: correlation with neuropsychological findings. Med Pediatr Oncol 2000;35:456-461.
118. Goldman ID, Matherly LH. The cellular pharmacology of methotrexate. Pharmacol Ther 1985;28:77-102.
119. Ulrich CM, Robien K, Sparks R. Pharmacogenetics and folate metabolism - a promising direction. Pharmacogenomics 2002;3:299-313.
120. Kraus JP, Oliveriusova J, Sokolova J et al. The human cystathionine B-Synthase (CBS) gene: complete sequence, alternative splicing, and polymorphisms. Genomics

1998;52:324.
121. Krull K, Brouwers P, Jain N et al. Folate pathway genetic polymorphisms are related to attention disorderes in childhood leukemia survivors. J Pediatr 2008;1:101-105.
122. Reddick WE, Shan ZY, Glass JO et al. Smaller white matter volumes are associated with larger deficits in attention and learning among long-term survivors of acute lymphoblastic leukemia. Cancer 2006;106:941-949.
123. Arico M, Valsecchi MG, Camitta B et al. Outcome of treatment in children with Philadelphia chromosome-positive acute lymphoblastic leukemia. N.Engl.J.Med. 2000;342:998-1006.
124. Druker BJ, Sawyers CL, Kantarjian H et al. Activity of a specific inhibitor of the BCR-ABL tyrosine kinase in the blast crisis of chronic myeloid leukemia and acute lymphoblastic leukemia with the Philadelphia chromosome 1. N.Engl.J.Med. 2001;344:1038-1042.
125. Ottmann OG, Druker BJ, Sawyers CL et al. A phase 2 study of imatinib in patients with relapsed or refractory Philadelphia chromosome-positive acute lymphoid leukemias. Blood 2002;100:1965-1971.
126. Scheuring UJ, Pfeifer H, Wassmann B et al. Early minimal residual disease (MRD) analysis during treatment of Philadelphia chromosome/Bcr-Abl-positive acute lymphoblastic leukemia with the Abl-tyrosine kinase inhibitor imatinib (STI571). Blood 2003;101:85-90.
127. Lee S, Kim DW, Kim YJ et al. Minimal residual disease-based role of imatinib as a first-line interim therapy prior to allogeneic stem cell transplantation in Philadelphia chromosome-positive acute lymphoblastic leukemia. Blood 2003;102:3068-3070.
128. Thomas DA, Faderl S, Cortes J et al. Treatment of Philadelphia chromosome-positive acute lymphocytic leukemia with hyper-CVAD and imatinib mesylate. Blood 2004;103:4396-4407.
129. Carter TA, Wodicka LM, Shah NP et al. Inhibition of drug-resistant mutants of ABL, KIT, and EGF receptor kinases. Proc.Natl.Acad.Sci.U.S.A 2005;102:11011-11016.
130. Shah NP, Tran C, Lee FY et al. Overriding imatinib resistance with a novel ABL kinase inhibitor. Science 2004;305:399-401.
131. Talpaz M, Shah NP, Kantarjian H et al. Dasatinib in imatinib-resistant Philadelphia chromosomepositive leukemias. N.Engl.J.Med. 2006;354:2531-2541.
132. Ottmann O, Dombret H, Martinelli G et al. Dasatinib induces rapid hematologic and cytogenetic responses in adult patients with Philadelphia chromosome-positive acute lymphoblastic leukemia with resistance or intolerance to imatinib: interim results of a Phase II study. Blood 2007blood- 2007.

133. Kantarjian H, Pasquini R, Hamerschlak N et al. Dasatinib or high-dose imatinib for chronic-phase chronic myeloid leukemia after failure of first-line imatinib: a randomized phase 2 trial. Blood 2007;109:5143-5150.
134. Hochhaus A, Kim DW, Rousselot P et al. Dasatinib (SPRYCEL(R)) 50mg or 70mg BID Versus 100mg or 140mg QD in Patients with Chronic Myeloid Leukemia in Chronic Phase (CML-CP) Resistant or Intolerant to Imatinib: Results of the CA180-034 Study. ASH Annual Meeting Abstracts 2006;108:166.
135. Kantarjian H, Ottmann O, Pasquini R et al. Dasatinib (SPRYCEL(R)) 140 mg Once Daily (QD) vs 70 mg Twice Daily (BID) in Patients (pts) with Advanced Phase Chonic Myeloid Leukemia (ABP-CML) or Ph(+) ALL Who Are Resistant or Intolerant to Imatinib (im): Results of the CA180-035 Study. ASH Annual Meeting Abstracts 2006;108:746.
136. Talpaz M, Shah NP, Kantarjian H et al. Dasatinib in imatinib-resistant Philadelphia chromosomepositive leukemias. N.Engl.J.Med. 2006;354:2531-2541.
137. Hilden JM, Dinndorf PA, Meerbaum SO et al. Analysis of prognostic factors of acute lymphoblastic leukemia in infants: report on CCG 1953 from the Children's Oncology Group. Blood 2006;108:441-451.
138. Jeha S, Gandhi V, Chan KW et al. Clofarabine, a novel nucleoside analog, is active in pediatric patients with advanced leukemia. Blood 2004;103:784-789.
139. Jeha S, Gaynon PS, Razzouk BI et al. Phase II study of clofarabine in pediatric patients with refractory or relapsed acute lymphoblastic leukemia. J.Clin.Oncol. 2006;24:1917-1923.
140. Hijiya N, Gaynon P, Barry E et al. A multi-center phase I study of clofarabine, etoposide and cyclophosphamide in combination in pediatric patients with refractory or relapsed acute leukemia. Leukemia 2009;23:2259-2264.
141. Ammann RA, Zucol F, Aebi C et al. Real-time broad-range PCR versus blood culture. A prospective pilot study in pediatric cancer patients with fever and neutropenia. Support.Care Cancer 2007;15:637-641.
142. Hakim H, Flynn PM, Knapp KM, Srivastava DK, Gaur AH. Etiology and Clinical Course of Febrile Neutropenia in Children With Cancer. J Pediatr.Hematol.Oncol 2009
143. Wayne, PA. Interpretive Criteria for Identification of Bacteria and Fungi by DNA Target Sequencing: Approved Guideline. CLSI document MM18-A. 2008. Ref Type: Generic
144. Chen Z, Plagemann PG. Detection of related positive-strand RNA virus genomes by reverse transcription/polymerase chain reaction using degenerate primers for common replicase sequences. Virus Res. 1995;39:365-375.
145. Coiras MT, Perez-Brena P, Garcia ML, Casas I. Simultaneous detection of influenza

A, B, and C viruses, respiratory syncytial virus, and adenoviruses in clinical samples by multiplex reverse transcription nested-PCR assay. J Med Virol. 2003;69:132-144.
146. Dyer J, Chisenhall DM, Mores CN. A multiplexed TaqMan assay for the detection of arthropodborne flaviviruses. J Virol.Methods 2007;145:9-13.
147. Einsele H, Hebart H, Roller G et al. Detection and identification of fungal pathogens in blood by using molecular probes. J Clin Microbiol. 1997;35:1353-1360.
148. Nix WA, Oberste MS, Pallansch MA. Sensitive, seminested PCR amplification of VP1 sequences for direct identification of all enterovirus serotypes from original clinical specimens. J Clin Microbiol. 2006;44:2698-2704.
149. VanDevanter DR, Warrener P, Bennett L et al. Detection and analysis of diverse herpesviral species by consensus primer PCR. J Clin Microbiol. 1996;34:1666-1671.
150. Seng P, Drancourt M, Gouriet F et al. Ongoing revolution in bacteriology: routine identification of bacteria by matrix-assisted laser desorption ionization time-of-flight mass spectrometry. Clin Infect.Dis. 2009;49:543-551.
151. Sjoholm MIL, Dillner J, Carlson J. Multiplex Detection of Human Herpesviruses from Archival Specimens by Using Matrix-Assisted Laser Desorption Ionization-Time of Flight Mass Spectrometry. J.Clin.Microbiol. 2008;46:540-545.
152. Varani S, Stanzani M, Paolucci M et al. Diagnosis of bloodstream infections in immunocompromised patients by real-time PCR. J Infect. 2009;58:346-351.
153. Xu J, Moore JE, Millar BC et al. Improved laboratory diagnosis of bacterial and fungal infections in patients with hematological malignancies using PCR and ribosomal RNA sequence analysis. Leuk.Lymphoma 2004;45:1637-1641.
154. Reiter A, Schrappe M, Ludwig WD et al. Chemotherapy in 998 unselected childhood acute lymphoblastic leukemia patients. Results and conclusions of the multicenter trial ALL-BFM 86. Blood 1994;84:3122-3133.
155. Hak LJ, Relling MV, Cheng C et al. Asparaginase pharmacodynamics differ by formulation among children with newly diagnosed acute lymphoblastic leukemia. Leukemia 2004;18:1072-1077.
156. Lugthart S, Cheok MH, den Boer ML et al. Identification of genes associated with chemotherapy crossresistance and treatment response in childhood acute lymphoblastic leukemia. Cancer Cell 2005;7:375-386.
157. Pieters R, Huismans DR, Loonen AH et al. Relation of cellular drug resistance to long-term clinical outcome in childhood acute lymphoblastic leukaemia. The Lancet 1991;338:399-403.
158. Leung W, Iyengar R, Turner V et al. Determinants of antileukemia effects of allogeneic NK cells. J.Immunol. 2004;172:644-650.
159. Leung W, Iyengar R, Triplett B et al. Comparison of killer Ig-like receptor

genotyping and phenotyping for selection of allogeneic blood stem cell donors. J.Immunol. 2005;174:6540-6545.
160. Basser PJ, Jones DK. Diffusion-tensor MRI:theory, experimental design and data analysis – a technical review. NMR Biomed 2002;15:456-467.
161. Bihan DLe, Mangin J-F, Poupon C et al. Diffusion tensor imaging: concepts and applications. J Magn Reson Imaging 2001;13:534-546.
162. Campell LK, Scaduto M, Sharp W et al. A meta-analysis of the neurocognitve sequelae of treatment for childhood acute lymphocytic leukemia. Pediatr Blood Cancer 2007;49:65-73.
163. Moleski M. Neuropsychological, neuroanatomical, and neurophysiological consequences of CNS chemotherapy for acute lymphoblastic leukemia. Arch Clin Neuropsychol 2000;15:603-630.
164. Bedell BJ, Narayana PA. Automatic removal of extrameningeal tissues from MR images of human brain. J Magn Reson Imaging 1996;6:939-943.
165. Mulhern RK, Wasserman AL, Fairclough D, Ochs J. Memory function in disease-free survivors of childhood acute lymphocytic leukemia given CNS prophylaxis with or without 1,800 cGy cranial irradiation. J Clin Oncol 1988;6:315-320.
166. Mulhern RK, Armstrong FD, Thompson S. Function-specific neuropsychological assessment. Med Pediatr Oncol 1998;Suppl 1:34-40.
167. Nathan PC, Patel SK, Dilley K et al. Guidelines for identification of, advocacy for, and intervention in neurocognitive problems in survivors of childhood cancer: a report from the Children's Oncology Group. Arch Pediatr Adolesc Med 2007;161:798-806.
168. Hockenberry M, Krull K, Moore K et al. Longitudinal evaluation of fine motor skills in children with leukemia. J Pediatr Hematol Oncol 2007;29:535-539.
169. Mullen EM. Mullen Scales of Early Learning.: Circle Pines: American Guidance Service, Inc.; 1995.
170. Nachman JB, Sather HN, Sensel MG et al. Augmented post-induction therapy for children with high-risk acute lymphoblastic leukemia and a slow response to initial therapy. N.Engl.J.Med. 1998;338:1663-1671.
171. Silverman LB, Gelber RD, Dalton VK et al. Improved outcome for children with acute lymphoblastic leukemia: results of Dana-Farber Consortium Protocol 91-01. Blood 2001;97:1211-1218.
172. Cheng C. Almost-sure uniform error bounds of general smooth estimators of quantile density functions. Statistics & Probability Letters 2002;59:183-194.
173. Kalbfleisch JD, Prentice RL. The Statistical Analysis of Failure Time Data. Hoboken, NJ: John Wiley & Sons, Inc.; 2002.

174. Gray, R. J. A class of K-sample tests for comparing the cumulative incidence of a competing risk. Ann Stat 16(3), 1141-1154. 1988. Ref Type: Journal (Full)
175. Fine, J. P. and Gray, R. J. A Proportional Hazards Model for the Subdistribution of a Competing Risk. J.Amer.Statistical Assoc. 94(446), 496-509. 1999. Ref Type: Journal (Full)
176. Bogni A, Cheng C, Liu W et al. Genome-wide approach to identify risk factors for therapyrelated myeloid leukemia. Leukemia 2006;20:239-246.
177. Flotho C, Coustan-Smith E, Pei D et al. Genes contributing to minimal residual disease in childhood acute lymphoblastic leukemia: prognostic significance of CASP8AP2. Blood 2006;108:1050-1057.
178. Holleman A, Cheok MH, den Boer ML et al. Gene-expression patterns in drug-resistant acute lymphoblastic leukemia cells and response to treatment. N.Engl.J.Med. 2004;351:533-542.
179. Silverman LB, Gelber RD, Dalton VK et al. Improved outcome for children with acute lymphoblastic leukemia: results of Dana-Farber Consortium Protocol 91-01. Blood 2001;97:1211-1218.
180. Cheok MH, Evans WE. Acute lymphoblastic leukaemia: a model for the pharmacogenomics of cancer therapy. Nat.Rev.Cancer 2006;6:117-129.
181. Relling MV, Pui CH, Cheng C, Evans WE. Thiopurine methyltransferase in acute lymphoblastic leukemia. Blood 2006;107:843-844.
182. Holleman A, Cheok MH, den Boer ML et al. Gene-expression patterns in drug-resistant acute lymphoblastic leukemia cells and response to treatment. N.Engl.J.Med. 2004;351:533-542.
183. Bogni A, Cheng C, Liu W et al. Genome-wide approach to identify risk factors for therapyrelated myeloid leukemia. Leukemia 2006;20:239-246.
184. Kishi S, Cheng C, French D et al. Ancestry and pharmacogenetics of antileukemic drug toxicity. Blood 2007;109:4151-4157.
185. Kishi S, Yang W, Boureau B et al. Effects of prednisone and genetic polymorphisms on etoposide disposition in children with acute lymphoblastic leukemia. Blood 2004;103:67-72.
186. Kishi S, Griener J, Cheng C et al. Homocysteine, Pharmacogenetics, and Neurotoxicity in Children With Leukemia. J Clin Oncol 2003;21:3084-3091.
187. Blanco JG, Edick MJ, Hancock ML et al. Genetic polymorphisms in CYP3A5, CYP3A4 and NQO1 in children who developed therapy-related myeloid malignancies. Pharmacogenetics 2002;12:605-611.
188. Relling MV, Yang W, Das S et al. Pharmacogenetic Risk Factors for Osteonecrosis of the Hip Among Children With Leukemia. J Clin Oncol 2004;22:3930-3936.
189. Mulligan CG, Goorha S, Radtke I et al. Genome-wide analysis of genetic

alterations in acute lymphoblastic leukaemia. Nature 2007;446:758-764.
190. Cheng Q, Yang W, Raimondi SC et al. Karyotypic abnormalities create discordance of germline genotype and cancer cell phenotypes. Nat.Genet. 2005;37:878-882.
191. Cheng C, Pounds SB, Boyett JM et al. Statistical significance threshold criteria for analysis of microarray gene expression data. Stat Appl.Genet.Mol.Biol. 2004;3:Article36.
192. Pounds S, Cheng C. Robust estimation of the false discovery rate. Bioinformatics. 2006;22:1979-1987.
193. Benjamini Y, Krieger AM, Yekutieli D. Adaptive linear step-up procedures that control the false discovery rate. Biometrika 2006;93:491-507.
194. Pounds S, Cheng C. Robust estimation of the false discovery rate. Bioinformatics 2006;22:1979-1987.
195. Pounds S, Cheng C. Sample size determination for the false discovery rate. Bioinformatics 2005;21:4263-4271.
196. Holleman A, Cheok MH, den Boer ML et al. Gene-expression patterns in drug-resistant acute lymphoblastic leukemia cells and response to treatment. N.Engl.J.Med. 2004;351:533-542.
197. Cheng C. Microarray gene co-expression analysis and validation by a sequential clustering algorithm [abstract]. Joint Statistical Meetings of the American Statistical Association 2007;Salt Lake City, UT:
198. De Pauw B, Walsh T, Donnelly J et al. Revised Definitions of Invasive Fungal Disease from the European Organization for Research and Treatment of Cancer/Invasive Fungal Infections Cooperative Group and the National Institute of Allergy and Infectious Diseases Mycoses Study Group (EORTC/MSG) Consensus Group. Clinical Infectious Diseases 2008;46:1813-1821.
199. Manabe A, Ohara A, Hasegawa D et al. Significance of the complete clearance of peripheral blasts after 7 days of prednisolone treatment in children with acute lymphoblastic leukemia: the Tokyo Children's Cancer Study Group Study L99-15. Haematologica 2008; 93:1155-1160.
